# Repository-based plasmid design

**DOI:** 10.1371/journal.pone.0223935

**Published:** 2020-01-09

**Authors:** Joshua J. Timmons, Doug Densmore

**Affiliations:** 1 Lattice Automation Inc., Boston, Massachusetts, United States of America; 2 Department of Electrical and Computer Engineering, Boston University, Boston, Massachusetts, United States of America; 3 Biological Design Center, Boston University, Boston, Massachusetts, United States of America; University of Helsinki, FINLAND

## Abstract

There was an explosion in the amount of commercially available DNA in sequence repositories over the last decade. The number of such plasmids increased from 12,000 to over 300,000 among three of the largest repositories: iGEM, Addgene, and DNASU. A challenge in biodesign remains how to use these and other repository-based sequences effectively, correctly, and seamlessly. This work describes an approach to plasmid design where a plasmid is specified as simply a DNA sequence or list of features. The proposed software then finds the most cost-effective combination of synthetic and PCR-prepared repository fragments to build the plasmid via Gibson assembly^®^. It finds existing DNA sequences in both user-specified and public DNA databases: iGEM, Addgene, and DNASU. Such a software application is introduced and characterized against all post-2005 iGEM composite parts and all Addgene vectors submitted in 2018 and found to reduce costs by 34% versus a purely synthetic plasmid design approach. The described software will improve current plasmid assembly workflows by shortening design times, improving build quality, and reducing costs.

## Introduction

Plasmids are a common design element in molecular biology. They are used for studying genes [1], encoding programs [2], and editing human cells [3]. Most are simple relative to their bacterial progenitors, containing just an origin of replication, resistance marker–combined together in a “backbone”–and an insert region of exogenous DNA [4]. But the exogenous region of plasmids are increasingly complex; genetic circuits in synthetic biology, for example, can comprise more than a dozen separate elements packaged together [2,5,6].

**Fig 1:**
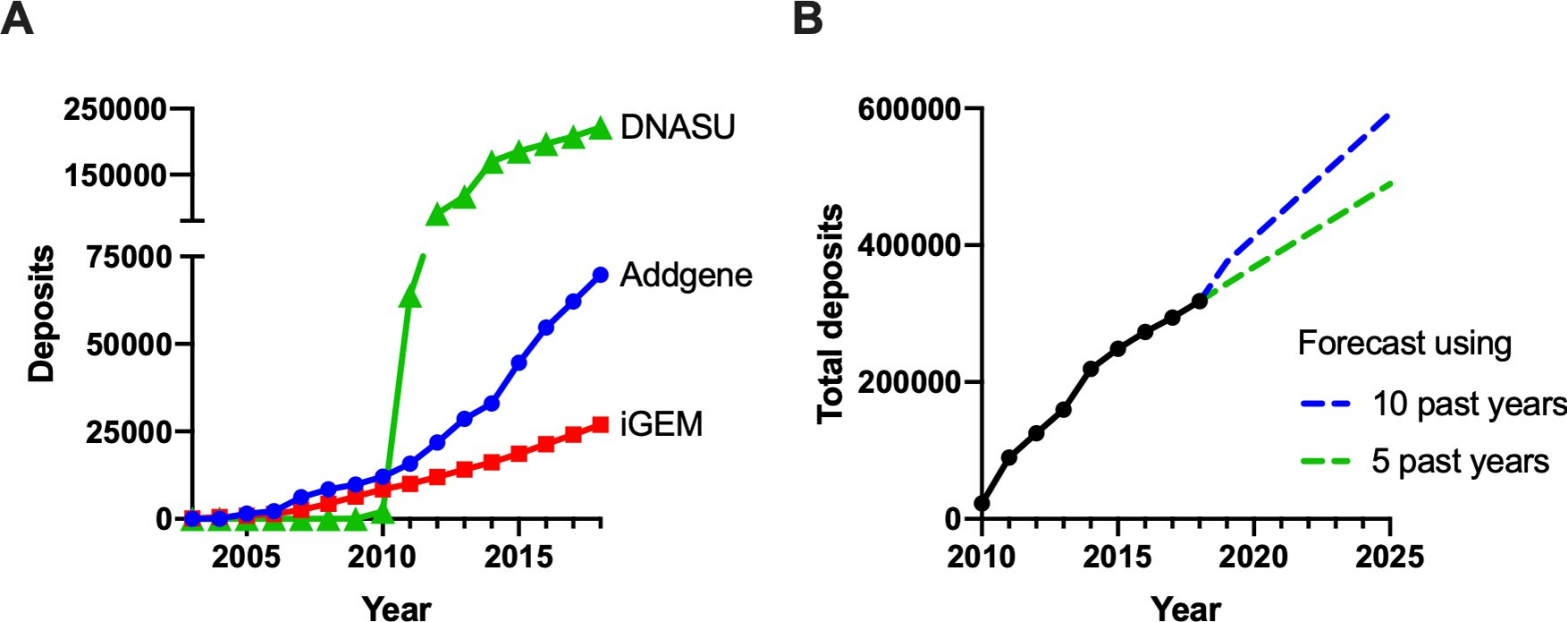
Rapid growth in DNA repository size. (A) Total number of DNA deposits in iGEM, Addgene, and DNASU from 2005 to 2018. (B) Total number of deposits between 2010 and 2018 with linear regression forecasts through 2025 using five (green) or ten (blue) years of past deposit totals. There was a 25-fold increase in the total number of sequence deposits available between 2008 and 2018 and a 99% increase between 2013 and 2018. Continued growth like the last five years would result in roughly 500,000 accumulated deposits between iGEM, Addgene, and DNASU by 2025.

Electronically available plasmid repositories are centers for plasmid sharing [7]. They enable inexpensive, reproducible, and collaborative experimental designs while handling the tedious processes of sample categorization and archiving [8]. Plus, they assist researchers required by journals to make their materials publicly available. And, whether by choice or pressure from journals and grant agencies, researchers have been submitting their plasmids to repositories in increasing numbers. Addgene grew from zero plasmids in 2004 to more than 70,000 in 2019 and has shipped over one million samples to researchers in 96 countries [8–10]. iGEM’s Registry of Standardized Biological Parts increased from 899 to more than 26,000 over the same time period [11,12] (Fig 1). DNASU now has greater than 200,000 plasmids and has shipped over 360,000 clones. Other large repositories include PlasmID [13], of Harvard Medical School, the BCCM/GeneCorner repository of Ghent University in Belgium, and the Fungal Genetics Stock Center [14] of Kansas State University. Plasmid repositories’ inventories will only grow in future years: another 100% increase, like the one that occurred over the last five years, would represent another 300,000 orderable plasmids.

When designing a new plasmid, an experimentalist can assemble it from fragments in their or their co-workers’ labs (“local” repositories) and public DNA repositories, or order it from a synthesis provider (either as a fragment or as a pre-cloned gene). Because combining fragments from local and remote repositories is presently less expensive than synthesis, and because plasmids in repositories may contain the features of biologists’ desired plasmids, we believe that plasmid repositories should be considered in the plasmid design process.

Repurposing DNA in repositories for new plasmids is non-trivial for several reasons. First, sequences in remote repositories are unstandardized–unlike those in the iGEM registry or Golden Gate kits [15–17]–so fragments will require preparation. Second, the researcher must be aware of where the redundant plasmid sequence is stored in remote repositories. Finally, even if a researcher is aware of fragments within a repository that they want to combine, they may fail to find the least expensive combination of repository fragments and synthesized fragments to assemble their plasmid without any off-target primers, replicated fragment junctions, or hairpins in the ends of synthetic fragments [18]. For example, the least expensive plasmid design for a user may contain a combination of fragments from Addgene, DNASU, and several short synthetic fragments. Finding and comparing all combinations of fragment sources from multiple repositories and synthesis providers, to build the least expensive plasmid, is beyond the capabilities of a human plasmid designer.

We have designed an application, REPP (an abbreviation of “repository-based plasmid”), that addresses the issues raised above. It collates sequences from both public and user repositories and factors in the cost of synthesis to find the least expensive plasmid design possible for Gibson assembly (circumventing the lack of standardization) [19–21]. It creates homology between adjacent fragments via primers (created with Primer3), and avoids primers with off-targets, replicated junctions, and synthetic fragments with hairpins in their ends.

REPP is open-source on Github [22] and pre-compiled binaries are available for Linux, MacOS, and Windows from SourceForge [23]. Primer3, BLAST, and BLAST databases for iGEM, DNASU, and Addgene are bundled with the application.

## Materials and methods

### Design overview

**Fig 2:**
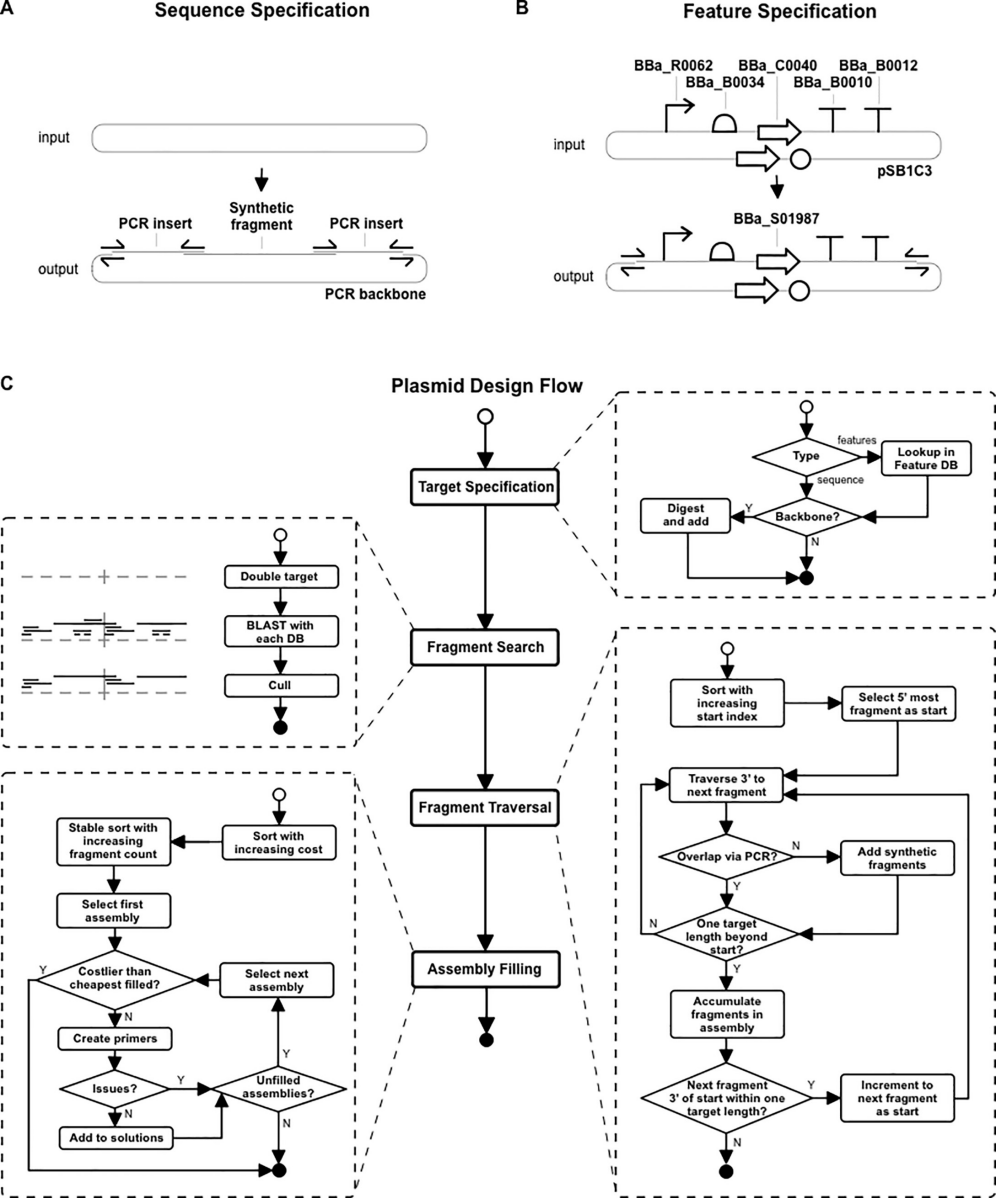
Flowchart of REPP’s plasmid design. (A) Sequence-specified plasmid design with an example of design output with one synthetic fragment and three PCR fragments. (B) Feature-specified plasmid design with an example where iGEM parts are ordered in the orientation of the target plasmid–REPP finds a vector, BBa_S01987, that matches the user’s design specification. Users can specify their target plasmid by its sequence, composite features, or composite fragments (not shown). (C) Flowchart with the macro-microflow of REPP. Unfilled circles are the start of each process and filled circles are the end. Rounded rectangles are tasks and diamonds are conditions. The flowchart highlights the four steps of REPP’s design: parsing the target plasmid, finding fragments to cover the target, traversing the fragments to create a list of assemblies, and filling the Pareto-optimal assemblies.

Users of REPP specify a target plasmid as either a DNA sequence or as a list of feature names. They also choose a set of fragment databases (BLAST databases) to use as build sources: Addgene, DNASU, and iGEM are included, and local (same machine) BLAST databases are supported as well. REPP then finds the minimum cost design instructions for Gibson assembly. It outputs the name and repository URL of build fragments used to construct portions of the target plasmid. It creates primers for amplifying the build fragments, with junctions for neighboring fragments, as well as a list of synthetic fragments if necessary. It chooses PCR fragments and synthetic fragments so the designs are the least expensive possible given the costs and constraints in a user specified configuration file. A high-level flow of REPP is detailed in Fig 2. For details about REPP’s implementation, we invite researchers to visit the application’s code repository.

### Repositories and databases

REPP, at the time of publication, has three internal fragment databases that correspond to three sequence repositories: iGEM [11], Addgene [24], and DNASU [25]. We downloaded the iGEM database from the iGEM website in XML format [26]. We received both Addgene and DNASU DNA sequences, upon request, in February 2019. The Addgene database was in JSON format and the DNASU database was in Excel. We parsed all the databases to multi-FASTA format and prepared each as a BLAST database [27].

If the user specifies a target plasmid using feature names, REPP looks up each in the Feature Database–a one to one mapping between name and sequence in tab-separated values (TSV) format. REPP comes with 1096 plasmid features made public by SnapGene (S3 Text) [28,29]. On selection in a feature-based plasmid specification, each feature sequence is individually queried against the user selected databases (with a default percentage identity of 96%). Users can also choose a backbone and enzyme. Without a backbone and enzyme, REPP assumes the target is a circular plasmid, but, with them, it digests the backbone with the enzyme, selects the largest digest fragment, and anneals the target DNA sequence to the backbone’s sequence. Backbones are selected by their ID in a database and enzymes are referenced by their name in REPP’s editable Enzyme Database–another map, this one between enzymes’ names and recognition sequences (also in TSV format, S3 Text). Both the Feature Database and the Enzyme Database are editable from REPP’s command line interface.

### Costs

Many distinct costs go into plasmid construction and REPP factors in several of them. The most complex and variable cost is that of synthesis. REPP’s user modifiable configuration file (Table A in S1 File), in YAML map format, supports a definition of synthesis cost curves with both variable (per basepair) and fixed costs. When REPP estimates the cost of a synthetic fragment, it looks up the first fragment integer key in the “synthetic-fragment-cost” map that exceeds the length of synthetic fragment. If the cost is fixed, it is used as is. Otherwise, the fragment’s length is multiplied by the cost (for a variable length cost). By default, the synthetic fragment cost curve and synthetic gene cost curve both correspond to IDT’s prices. Primer cost is also factored in with a default price of $0.60 per bp (IDT). Taq DNA Polymerase PCR Buffer is included at $0.27 per reaction (ThermoFisher). Gibson assembly Master Mix is included and is estimated at $12.98 per assembly (NEB). Additionally, the cost of DNA procurement from iGEM, Addgene, and DNASU is estimated at $0, $65, and $55 per source plasmid. REPP does not account for less expensive bulk order costs from Addgene or DNASU. REPP also does not account for Addgene’s more limited selection of plasmids available to scientists at for-profit organizations (users must email the plasmid’s depositing lab directly). Further, iGEM costs are ignored by REPP because they are bundled in iGEM teams’ registration costs or paid for once with a yearly “Labs Program” subscription [30]. It assumes a $0 procurement cost for fragments in a user-defined repository.

An interesting but highly variable cost is that of “human hours”: the cost of a researcher’s time to assemble the plasmid after PCR and Gibson assembly. There are two configurable human hour costs in the configuration file: one for the time spent on PCR amplification of the fragments and another for time spent on the Gibson assembly. Both are zero by default.

### Fragment search

REPP finds building fragments using the target plasmid sequence doubled, end to end, as a query against each of the user selected databases. BLAST’s “blastn” task is used with reward, penalty, gapopen, and gapextend values based upon the researcher’s specified percentage identity. For example, at a percentage identity of 90% REPP uses a reward, penalty, gapopen and gapextend of 1, -2, 1, and 2, respectively. These values are documented in Table B in S1 File and are based largely upon the BLAST user manual recommendations [31]. The one exception is penalty, gapopen, and gapextend values of -6, 6, and 6 when the user specifies a percentage identity greater than 99%–we found that, to avoid imperfect hits and mismatches at the end of fragments, we needed to supply penalties that exceeded typical values for these parameters. After fragment matches are found and sorted along the sequence, we cull the matches to remove fragments that are fully engulfed by others. Our algorithm’s runtime is bound by BLAST and the Fragment Search step.

### Fragment traversal

Fragments that are within one target plasmid sequence length of the start become seed points for plasmid traversal along target plasmid sequence. Candidate assemblies are accumulated during a 5’ to 3’ traversal along the target sequence from each seed, starting point. During traversal, each fragment is sequentially “annealed” into candidate assemblies with each fragment to its 3’ end. If there is a gap between fragments, the estimated number of synthetic fragments necessary to fill it is estimated. If an assembly is generated that spans the entire length of the target sequence, and has fewer fragments than the configured limit, it is stored as a possible assembly for assembly filling.

### Assembly filling

REPP’s two goals when constructing a plasmid are to minimize the design’s cost and total number of fragments. These goals usually conflict. Generally, a plasmid with large and synthetic fragments will be more expensive but require less work and preparation than a plasmid with many more fragments prepared with PCR. Because of this trade-off, REPP returns the Pareto optimal set of solutions for each target plasmid so the user can choose for themselves. During assembly filling, it sorts assemblies in ascending order first by their cost and then by their number of fragments. It does so to avoid non-Pareto optimal plasmid designs. If a hypothetical assembly has four fragments, but costs more than an earlier assembly that was filled with only three fragments, REPP skips the remaining assemblies to reduce execution time. The trade off in lower fragment counts for higher costs is apparent in Fig B in S1 File where, when REPP designs multiple solutions, those with only two fragments are 1.7 times more expensive than the lease expensive solution, on average. Other than this paragraph, when describing a plasmid’s “solution,” we are referring to the solution with the lowest cost, ignoring that REPP may have returned other more expensive solutions with fewer fragments.

When filling an assembly, REPP creates primers for the repository-derived fragments using Primer3 [32,33]. By default, neighboring fragments must have at least 15 bp of homology for one another and no more than 150 bp. If adjacent fragments in an assembly will both be PCR amplified and both lack homology for one another in their source material, additional bp are included within primers’ sequences–the additional bp are equally distributed between the adjacent fragments. Conversely, when two fragments have excessive homology for one another REPP uses a subsequence one or both of the fragments to reduce the total length of overlap (Fig B in S1 File). If there is more than the minimum amount of homology in a junction between fragments, or if one of the fragments is synthetic and the other is the product of PCR, Primer3 is given a range on the fragment’s source material so it can choose an “optimal” primer pair with more flexibility. Finally, if there is a small “gap” between two neighboring fragments that will be PCR amplified before Gibson assembly, the template sequence will be embedded within the fragments’ primers. The default maximum length for primer-filled sequence gaps is 20 bp.

REPP checks primers for ectopic, mismatching binding sites in building fragments’ source sequences. It does so with an approach based upon Primer-BLAST [34]. Primer sequences are queried with BLAST using a percentage identity of 65% and an expected value cutoff of 30,000. The annealing tm of each potential ectopic binding site found through BLAST is estimated with Primer3’s “ntthal” and “END1” mode where the first sequence is the primer and the second sequence is the reverse complement of the ectopic sequence. Assemblies are not filled if they include primers with an ectopic binding site with a primer melting temperature exceeding REPP’s “pcr-primer-max-ectopic-tm” parameter.

REPP also generates synthetic fragments during the fill step. For spans larger than the maximum synthetic fragment size limit, it splits the region into sub-fragments and creates each individually. It checks for hairpins in the junctions between the sub-fragments using “ntthal”‘s hairpin mode. If it predicts that a hairpin will form at the end of a sub-fragment and that it will exceed the “fragments-max-hairpin-tm” parameter in the configuration file, it iteratively extends the 3’ end repeatedly in an attempt to avoid it.

### Characterization with iGEM and Addgene datasets

To test REPP we used it to construct two DNA sequence datasets: one from iGEM and one from Addgene [35,36]. We specified the target plasmids’ sequences, and REPP output the repository fragments and primers to generate each plasmid’s sequence. The synthesis provider for both iGEM and Addgene datasets was IDT. For both runs, the source repository was the sole source of building fragments; only iGEM was used to construct the iGEM dataset and only Addgene was used to construct the Addgene dataset. When building each dataset, we filtered out fragments from “future” years in the repository. For example, when we built a plasmid with a composite part from 2008, we excluded all iGEM parts from 2008 through present day to mirror the tool’s imagined utility to a user in 2008. Similar filtering functionality is available via an “exclude” flag in REPP’s command line interface.

The iGEM dataset (S2 File) contained all 24,909 parts submitted between 2005 –the year after pSB1A3 was submitted–and 2018 [11,12]. We assembled each in plasmids with the backbone pSB1A3 linearized with PstI [37]. The iGEM repository was the most attractive test dataset–among iGEM, Addgene, and DNASU–because all its parts can be used as insert sequences in new plasmid designs. Knowing each plasmid’s insert sequence allowed us to compare REPP’s specified assembly costs against synthesis options.

The Addgene dataset (S3 File) contained the 7,352 plasmids uploaded to Addgene in 2018. Unlike the iGEM dataset, it was not explicit which parts of the plasmid were backbone versus insert so we could not compare the solutions’ costs to synthesis providers.

To test REPP’s performance with multiple synthesis providers, we created separate settings files with synthetic fragment cost curves from six synthesis providers, as of February 2019 (S1 Text and Fig A in S1 File), and rebuilt both datasets. Every REPP plasmid design solution was checked for validity by programmatically comparing the ends of adjacent fragments in the designs. If there was at least 15 and no more than 150 bp of overlap between the end of each fragment and the one after it a junction was considered valid. Both datasets were built with REPP on a Google Compute instance with 16 virtual cores and Ubuntu 16.

### Characterization with As0

To further characterize REPP, we used it design a recently submitted (2019) plasmid, As0 (Addgene #123158), containing a bacterial arsenic sensor [38]. Because of its recent submission date, As0 was not part of REPP’s embedded Addgene plasmid database so it could not be used as a solution without modification (as is generally the case for near-exact target matches). The sequence file for Addgene #123158 was downloaded in Genbank format and was used as the input target sequence for REPP’s “make sequence” command. Twist synthesis prices (S1 Text) were used and only Addgene was used as a source repository. REPP’s output payload, in JSON format, was converted to an Excel file (S4 File) and its generated PCR fragments, PCR primers, and synthetic fragments were overlaid upon the original As0 plasmid map in SnapGene 5.0’s GenBank format (S5 File).

### Statistical analysis

To investigate whether synthesis provider’s costs and selected percentage identity affected the cost of plasmid design prices within the iGEM and Addgene datasets, data were analyzed via GraphPad Prism 8.3 (Fig 4). Two-tailed nonparametric Mann-Whitney *U* tests were used to make comparisons between synthesis providers (IDT versus Twist Biosciences) and percentage identities (100% versus 98%; 98% versus 96%). *P* values of 0.05, 0.01, 0.001, and 0.0001 were used for levels of significance.

## Results

After building iGEM plasmids with DNA fragments from the iGEM repository and/or synthetic fragments, we estimated the total assembly costs to build the iGEM dataset via REPP, synthetic fragments, and synthetic genes to be $3,594,327, $5,411,060 and $12,854,997. In other words, if the entire dataset was assembled, REPP would save 34% versus synthetic fragments alone and 72% versus synthetic genes. Approximately 35% of plasmids had at least 30% cost savings compared to synthetic fragments alone (Fig 2C).

**Fig 3:**
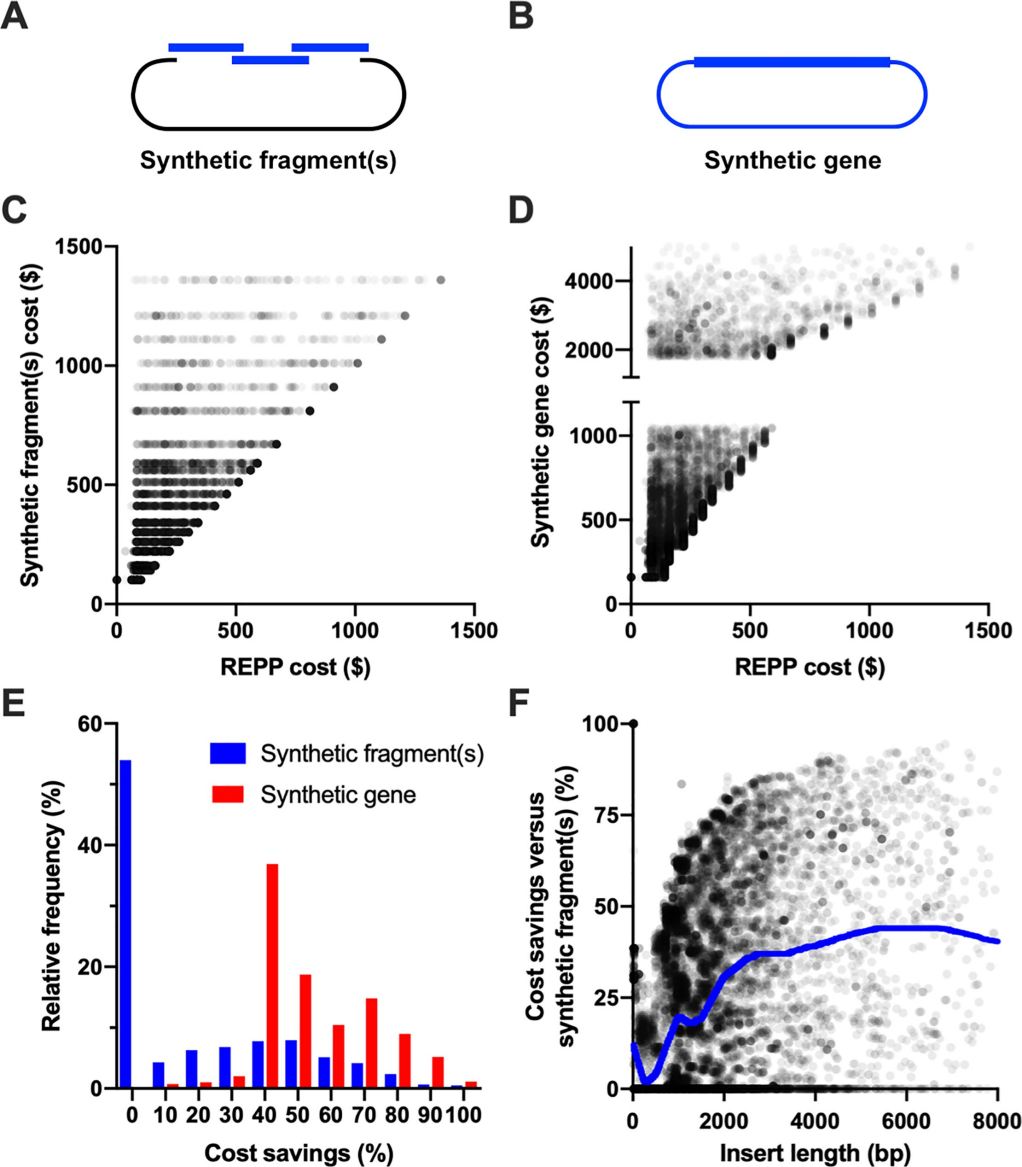
Plasmid design of iGEM plasmids from preexisting iGEM parts. REPP’s design of iGEM composite devices with 98% identity, synthesis with IDT, and iGEM itself as the sole fragment source repository. (A) An alternative to REPP’s designs of each plasmid was their assembly with synthetic fragment(s) alone without PCR amplified sub-fragments. If an iGEM part was too long for a single synthetic fragment, it was divided into multiple synthetic sub-fragments. (B) Another alternative build approach was ordering the iGEM part in a “synthetic gene” where the part is delivered in a pre-cloned plasmid. (C) Cost comparison between plasmids designed with synthetic fragment(s) inserted into linearized pSB1A3 and REPP’s plasmid designs for all iGEM plasmids. For the top quartile of the plasmid designs, REPP’s solution was at least 39% less expensive than synthetic fragments alone. (D) Similar cost comparison between REPP’s plasmid designs and synthetic gene costs. REPP’s solution was always the same cost or less expensive, though often included only synthetic fragments. (E) Frequency distributions for the cost savings of REPP’s plasmid designs versus synthetic fragment(s) (blue) and synthetic genes (red). (F) Length of the iGEM part (insert) versus REPP’s cost savings against synthetic fragment-only plasmid design. A Lowess curve, blue, was created with a windows size of 15%. REPP’s savings were greater than 25% for all plasmid designs greater than 2,000bp and averaged around 37% for plasmids with 3,000bp iGEM parts.

The length of the inserted iGEM part greatly impacted cost savings (Fig 3F). While REPP reverted to synthesis for almost all small plasmids less than 500bp, its savings versus synthetic fragments alone improved to an average of at least 37% for all plasmids with iGEM parts longer than 3,000bp. This makes intuitive sense: there were more opportunities for sequence re-use from existing parts in the iGEM repository.

After building the Addgene dataset, the median cost for Addgene plasmid designs for Addgene was $374 versus $155 for the iGEM dataset. The difference can be explained by the standard linearized backbone (pSB1A3) used for the iGEM datasets–without it in the Addgene dataset, REPP had a greater amount of variable sequence to cover in each plasmid.

Choice in synthesis provider affected REPP’s solution costs. For the iGEM dataset, the plasmid cost quartiles were $65, $85, and $125 versus $102, $162, and $233 from Twist Bioscience and IDT, respectively (Fig 3A; P<0.0001). It should be noted that REPP does not, yet, encode or check for synthesis complexities from different synthesis providers. The probability and time of procurement may vary between synthesis providers but was not factored into costs or plasmid designs. There are other tools, like BOOST, that support sequence redesigns to improve synthesis favorability [18,39].

**Fig 4:**
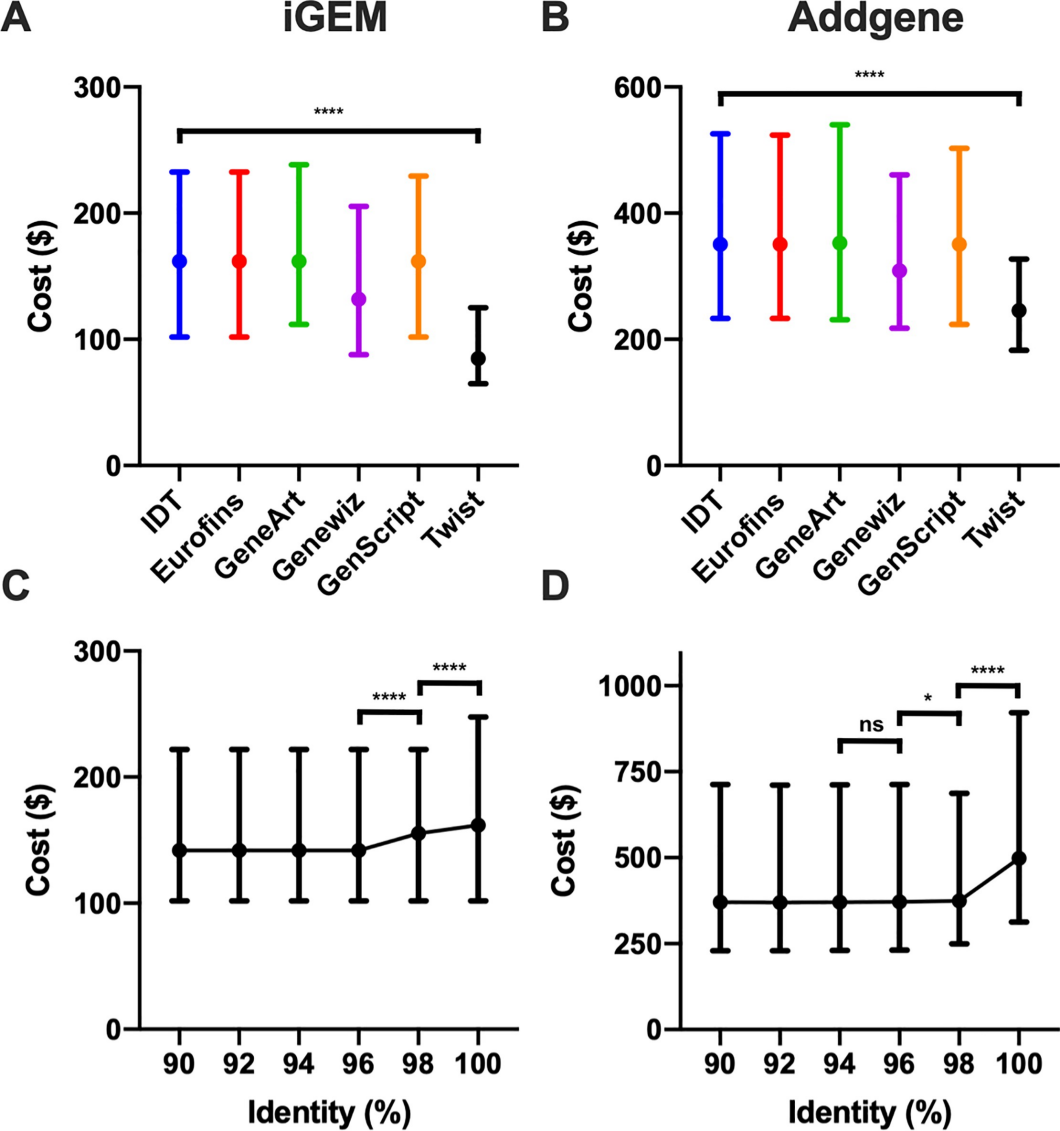
Cost sensitivities. Plasmid assembly costs for the iGEM and Addgene datasets in response to varied synthesis providers and percentage identities. Median and interquartile range of REPP designed plasmid costs using synthesis costs corresponding to IDT, Eurofins, GeneArt, Genewiz, GenScript, and Twist Bioscience for the iGEM (A, C) and Addgene (B, D) datasets. Synthesis costs were from February 2019. Less expensive synthesis costs like Twist Bioscience’s corresponded with less expensive plasmid designs. REPP’s specification file makes synthesis costs adaptable to future price changes. In (A) the dataset is the same as Fig 3: composite iGEM parts inserted into pSB1A3 digested with PstI and a 98% sequence identity requirement. The plasmids in (B, D) were all uploaded to the Addgene repository in 2018. Median and interquartile range of plasmid costs in response to varied percentage identities for the iGEM (C) and Addgene 2018 (D) datasets. Two-tailed Mann-Whitney *U* test was applied (*ns* ≥ 0.05, *p < 0.05, ****p < 0.0001).

Percentage identities less than 100% led to less expensive plasmid designs (Fig 4C and 4D). The mean price declined from $222 to $206 for iGEM and from $695 to $540 for Addgene when the percentage identity of the target plasmid was lowered from 100% to 98% (P<0.0001). A lower identity, with its concomitant loosening of BLAST parameters, led to REPP finding a greater number of potential building fragments and less expensive overall solutions. Because of the large drop-off in cost at 98% identity–and the smaller drop-off in median cost at 96% (Fig 4D)–we used 98% as the default percentage identity. Percentage identity is adjustable through REPP’s CLI.

To demonstrate REPP on a recent plasmid in the literature, we used it to design an assembly for As0: an arsenic sensing plasmid that was submitted to Addgene in mid-2019 (Addgene #123158) [38]. REPP designed As0 with two PCR fragments (2,229 bp and 4,223 bp) interleaved by two synthetic fragments (766 bp and 812 bp). Because the PCR fragments’ (Addgene #78637 and Addgene #61439) depositing lab was the same as the target plasmid (As0; Addgene #123158), we set their procurement costs to zero dollars. Given the costs of primer and synthetic fragment synthesis, as well as Gibson assembly and PCR reactions, we estimated the plasmid’s total cost to be $176. Files for the fragments and primers in spreadsheet format (S4 File) and plasmid map format (S5 File) are in Supporting Information.

REPP’s median build time for the iGEM dataset was 0.46 ± 1.4 seconds while the median build time for the Addgene dataset was 15 ± 8.9 seconds; BLAST was the bottleneck in execution times for both datasets.

## Discussion

We have demonstrated that plasmid design benefits from pairing with DNA repositories. We have made and characterized an open-source application, REPP, that creates Gibson assembly fragments by re-using DNA within public and user-specific DNA repositories. It finds sub-fragments, creates primers for their amplification, and supports synthesis so the least expensive set of fragments are generated for a particular assembly.

In our investigation of REPP’s performance constructing iGEM and Addgene datasets–with synthesis as a point of comparison–we found that REPP performs best on plasmids with inserts greater than 1,000bp for which the probability of sequence re-use is greatest. Its cost savings versus synthetic fragments alone improved to an average of at least 37% for all plasmids with iGEM parts longer than 3,000bp. We note that in this characterization the cost of labor for PCR and Gibson assembly were set to zero; only the cost of reagents, primer synthesis, and fragment synthesis were included. In reality, there is an opportunity cost associated with time spent on DNA assembly. We left these costs unspecified because the human cost of a PCR or Gibson assembly reaction is highly variable and depends on the lab’s country, expertise, and throughput. The cost of each Gibson assembly and batched PCR, with regards to human time, can be specified within REPP’s configuration file (as described in S1 File).

We also used REPP to design a plasmid recently submitted to Addgene, As0 [38]. REPP recommended PCR-based re-use of the plasmid’s Ampicillin resistant backbone and coding regions of the HrpRS-based amplifier; these regions accounted for over 80% of the plasmid’s total sequence. REPP recommended synthesis of the *P*_*rinA_p80α*_ promoter and *rinA_p80α* gene (first synthetic fragment) and the promoter and ribosome binding site that drive expression of *arsR* (second synthetic fragment) (S4 File and S5 File). These two regions were, as reported in *Wan et al*. *2019* [38], created by synthesis and replacement via PCR amplification, respectively, indicating similarity between REPP’s design the original.

There are other tools for preparing a list of DNA fragments for Gibson assembly. They include, but are not limited to, j5 [40], Benchling [41], SnapGene [28], and Geneious [42]. Each simulates an assembly of fragments and outputs a list of fragments with primers for preparation. But each depends on the user knowing beforehand which combination of fragments they want in the plasmid design–despite a less expensive solution possibly existing in the freezer or among the plasmids in external repositories. REPP incorporates user repositories, public repositories, and synthesis–applying costs to each–to build minimum cost solutions that abide by Gibson assembly design rules.

REPP reverses the design process of the aforementioned plasmid design tools. Rather than choosing fragments and annealing them into a plasmid, the user specifies a plasmid’s sequence or features and REPP decomposes it into the least expensive set of fragments and primers. In software engineering terms, REPP has a declarative interface rather than an imperative one [43]; users specify the plasmid they want rather than the fragments to put it together. We believe this higher-level approach to plasmid design will be more intuitive and powerful for users today and automated labs of the future where sequences are generated automatically without human intervention. REPP includes fragment-based plasmid specification as well, but it is not discussed here.

A recently released but related tool is DNA Weaver [44]. Like the tool discussed here, it accepts a “target” DNA sequence as an input and outputs the fragments, existing or synthetic, to assemble it. The greatest difference between DNA Weaver and REPP is that DNA Weaver constructs linear sequences while REPP builds circular plasmids. As a consequence, where DNA Weaver has a pre-defined start and end, matching those of the target sequence, REPP uses many fragment “seeds” as starting points for fragment traversal around the circular target plasmid’s sequence. For this reason, the applications’ outputs are not directly comparable; the cost-optimal set of fragments for a linear sequence may be suboptimal when it is circular or an insert within a plasmid and vice versa. Other differences between REPP and DNA Weaver include REPP’s feature-based plasmid design and embedded sequence repositories, which are absent in DNA Weaver, and DNA Weaver’s graphical build reports and Golden Gate assembly support, which are unsupported by REPP.

REPP’s scope could be expanded in the future. First, the approach described here is specific to Gibson assembly but could be adapted for other cloning methods. For example, sets of building fragments that have the requisite fusion sites for a complete Golden Gate assembly could be used without modification; otherwise, fragments could be prepared for Golden Gate via primers with embedded BsaI or BbsI sites [45]. Second, genomes and additional sequence repositories could augment REPP’s list of build fragment sources via a remote database management system like SynBioHub [46]–users could specify the genome or public repository they would like to include as a build source and those sources would be downloaded on command. Third, REPP could be expanded to include additional metadata from synthesis providers’ web-APIs like time-to-build as well as sequence complexities that limit the feasibly of synthesis–both would further inform biologists’ decision to synthesize or PCR amplify their assembly’s fragments. Finally, REPP’s bio-design capabilities could be augmented via pairing with higher-level tools like Eugene [47], Cello [2], and Double Dutch [48].

We believe that the sequence and feature based plasmid design approaches described here are more intuitive that existing state-of-the-art fragment-based design tools and will enable complex and multi-source plasmid designs that are otherwise infeasible.

## Supporting information

S1 File Supplementary information. Configuration documentation, synthesis cost curves, explanation of primer creation conditions, and example input and output.
(DOCX)

S2 File iGEM dataset. Multi-FASTA of iGEM target plasmids that were each constructed by REPP. Each plasmids’ iGEM name and year of submission is included.
(ZIP)

S3 File Addgene dataset. Multi-FASTA of Addgene target plasmids that were constructed by REPP. Each plasmid’s Addgene ID is included.
(ZIP)

S4 File REPP design example (Spreadsheet). Excel table with REPP generated PCR fragments, primers, and synthetic fragments to create As0 plasmid (Addgene #123158).
(XLSX)

S5 File REPP design example (Map). Genbank of REPP Design from S4 File: an annotated As0 plasmid overlaid with features corresponding to the fragments generated by REPP.
(GBK)

S1 Text Synthesis costs. A list of synthesis costs for Eurofins, GeneArt, GeneWiz, GenSript, IDT, and Twist Bioscience.
(TXT)

S2 Text REPP restriction enzymes. Default REPP restriction enzymes.
(TXT)

S3 Text REPP features. Default REPP features.
(TSV)
