# Correction: Repository-based plasmid design

**DOI:** 10.1371/journal.pone.0229981

**Published:** 2020-02-27

**Authors:** Joshua J. Timmons, Douglas Densmore

The second author’s name is spelled incorrectly. The correct name is: Douglas Densmore.

The ORCID iD is missing for Douglas Densmore. Please see their ORCID iD here:

- Author Douglas Densmore’s ORCID iD is: 0000-0002-7666-6808 (https://orcid.org/0000-0002-7666-6808).
