# Supplementary material for: Repository-based plasmid design
Source: PLoS One. 2020 Jan 9;15(1):e0223935. doi: 10.1371/journal.pone.0223935 (PMC6952187; doi:10.1371/journal.pone.0223935)
Supplement: S1 File — Configuration documentation, synthesis cost curves, explanation of primer creation conditions, and example input and output. (DOCX) [file pone.0223935.s001.docx]

**Repository-based plasmid design**

**Supplemental Information**

1. Configuration
   1. Application settings
   2. BLAST parameters
   3. Synthesis provider costs
   4. Enzymes database
   5. Feature database
   6. Primer creation conditions
2. Commands
   1. Sequence plasmid specification
   2. Feature plasmid specification
   3. Fragment plasmid specification
   4. Finding fragments/features/enzymes
   5. Setting feature/enzymes
3. Test
   1. iGEM
      1. Input
      2. Output
      3. Part count versus cost
   2. Addgene
      1. Input
      2. Output

**1. Configuration**

**1.1. Application Settings**

Most design parameters in REPP are configurable through the YAML settings file. A list of the names, meanings, and default values of the parameters is below. The parameters are configurable either by editing the default configuration file directly (config.yaml) or via reference to another YAML settings file through the “--settings” flag during plasmid specification. During a build step where a custom settings file is referenced through the “--settings” flag, all user defined settings overwrite those of the default configuration file.

***Supplemental Table A: REPP Settings***

| fragments-max-count | 6 | Maximum number of fragments allowed in a plasmid design. Larger numbers of fragments limit assembly efficiency ^1^. |
| --- | --- | --- |
| fragments-min-junction-length | 15 | Minimum length of bp overlap between adjacent fragments. |
| fragments-max-junction-length | 120 | Maximum length of bp overlap between adjacent fragments. |
| fragments-max-junction-hairpin | 47.0 | Maximum annealing temperature allowed in primers and at the ends of synthetic fragments. ^2^ |
| gibson-assembly-cost­ | 12.98 | The per reaction dollar cost of each Gibon Assembly reaction. Based upon the per reaction cost of NEB’s Gibson Assembly Master Mix. ^3^ |
| gibson-assembly-time-cost | 0.0 | The per reaction cost of human hours for the assembly. Depends on researcher’s value of time and the length required per assembly. |
| pcr-bp-cost | 0.6 | The per bp cost of each primer bp. Used in estimating the final assembly cost of each assembly. Cost is based upon IDT’s primer bp cost for 100nmol of single-stranded DNA as of February 2019 ^4^. |
| pcr-rxn-cost | 0.27 | The per reaction cost of PCR. Estimated using the per reaction cost of ThermoFisher’s Taq DNA Polymerase PCR Buffer (10X) ^5^. |
| pcr-time-cost | 0.0 | The per reaction of human time for each PCR reaction. This cost is applied across each assembly. So an $85 human cost for a PCR assembly include all PCRs necessary for that assembly. |
| pcr-min-length | 60 | The minimum number of bp necessary for a fragment to be PCR’ed. Fragment matches less than this length are not considered. |
| pcr-primer-max-pair-penalty | 30.0 | The maximum pair penalty for primers generated via Primer3. The configuration penalty is related to Primer3’s PRIMER_PAIR_*_PENALTY score and is used to filter out poor primer combinations with large mismatches in annealing temperature or heterodimers. ^2^ |
| pcr-primer-max-embed-length | 20 | The maximum length of embedded sequence at the end of a fragment via mutation in a primer. |
| pcr-primer-max-ectopic-tm | 55.0 | The maximum tolerable primer annealing temperature against an ectopic binding site. Calculated via the “ntthal” binary in Primer3. ^2^ PCR products with primers whose ectopic binding tm exceed this value are ignored. |
| pcr-buffer-length | 20 | The allowable range in which REPP lets Primer3 optimize primer pairs. Used when a PCR fragments neighbor is synthetic. The synthetic fragment can be expanded to overlap whatever range the PCR fragment winds up spanning, so Primer3 is given a range in which to generate primer pairs, rather than a fixed start. |
| synthetic-min-length | 125 | The minimum length of a fragment to be considered or synthesized. |
| synthetic-max-length | 3000 | The maximum length of a fragment to be considered for synthesis. Synthetic spans of DNA larger than this are fragmented into smaller synthetic fragments with overlap for one another. |
| synthetic-fragment-cost | map | A map from the maximum allowable synthetic length to the cost to make a synthetic fragment up to that length. The key is the maximum length (integer) and there are two values: fixed (bool) and cost (float). If the cost is fixed, that cost is used. If the cost is not fixed, the estimated cost of the synthetic fragment is the cost per bp multiplied by the synthetic fragment’s length. Example in section 1.3. The default cost map corresponds to the cost of IDT’s gBlocks as of February 2019 ^6^. |
| synthetic-plasmid-cost | map | Similar cost structure to synthetic-fragment-cost. Costs correspond to IDT’s “Custom gene synthesis” service as of February 2019 ^7^. |
| addgene-cost | 65.0 | The cost of procuring a plasmid from Addgene ^8^. |
| igem-cost | 0.0 | The cost of procuring an iGEM part from iGEM ^9^. |
| dnasu-cost | 55.0 | The cost of procuring a plasmid from DNASU ^10^. |

**1.2. BLAST parameters**

BLAST is used to find subsequences of plasmids that match existing sequences in public or user fragment databases. *blastn is* executed with a reward, penalty, gapopen, and gapextend that depend on the user defined percentage identity, specified below. The percentage identity parameter is configurable through the “identity” flag of plasmid make commands and has a default value of 98%. With the exception of the very strict parameters at >99% identity, these values are based on the BLAST User Manual ^11^.

***Supplemental Table B: BLAST Parameters***

| Identity (%) | reward | penalty | gapopen | gapextend |
| --- | --- | --- | --- | --- |
| > 99 | 1 | -5 | 6 | 6 |
| >= 98 | 1 | -3 | 3 | 3 |
| > 90 | 1 | -2 | 1 | 2 |
| - 0 | 1 | -1 | 1 | 2 |

Additionally, the flag “num_threads” is used with a value that is one less than the number of logical cores available to the process – unless the number of available logical cores is one, in which case one is used.

**1.3. Synthesis costs**


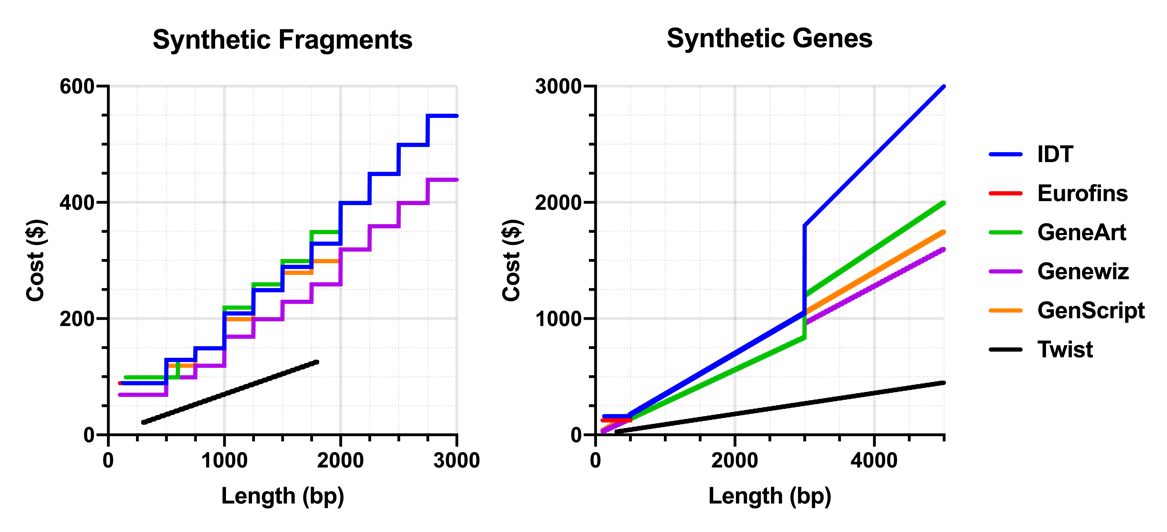


***Supplemental Figure A: Synthesis Cost Curves***

*Cost curves from six major synthesis providers, in February 2018, for synthetic fragments and synthetic genes. Cost curves were codified in separate configuration files and provided to REPP through its “settings” flag. The costs of the resulting assemblies were compared (Figure 4).*

Full cost curves for each provider, as were provided in YAML format, are available in “supp1_synthesis_costs.txt.”

**1.4. Enzyme database**

Users can provide either a full circular plasmid representing the full plasmid to make, or they can provide a linear sequence of DNA – the insert DNA – and specify a backbone and enzyme. If a backbone and enzyme are specified, the backbone is retrieved from the first match found among the fragment databases, and the enzyme is looked up in the enzyme database. The enzyme is then used to simulate a digest of the backbone and the insert DNA is inserted into the cleaved backbone to re-linearize it. The first matched recognition sequence of the enzyme is used to cleave the backbone.

Enzymes were collected from a New England Biolabs ^12^ webpage in February 2019 and parsed into to tab separated variable file. The first column has enzyme names and the second has the recognition sequence and cutsite of the sequence and complement sequence marked with “^” and “_”, respectively.

Commands for finding and editing the enzymes in the enzyme database are documented below. The full file is available in “supp2_enzymes.txt.” Examples enzyme are below:

AscI GG^CGCG_CC

AsiSI GCG_AT^CGC

FseI GG_CCGG^CC

NotI GC^GGCC_GC

PacI TTA_AT^TAA

**1.5. Feature database**

One of REPP’s supported plasmid specifications is what we call feature-based plasmid specification. Users specify a list of features, by name, along with their orientation. More documentation on feature-based plasmid specification is below.

Features were accumulated from a list of plasmid features published by SnapGene ^13^. The features were downloaded in SnapGene format and parsed to name and sequence key value pairs. Repeat features were removed and the features were parsed to a tab separated variable file in the same format as the enzyme database: the first column has names and the second has sequences. The raw features files are available upon request and the script used to parse the SnapGene files to a tab separated variable files is available in REPP’s Github repository in “scripts/snapgene_features_parse.py.”

Commands for finding and editing the features in the feature database are documented below. The full feature database is available in “supp3_features.tsv”. Two example features are below:

aspartic endopeptidase ATGTTTGCACCACAAGGCTTAGCTCAATTTATAAAAGTCAATGTAACCCTA

GAAAATGGTGAGCCCGTATTTATTTATACCGATGCCAATGGTCAAGTGTGCCAAGGTGATATCACCGTCACTCAAGCTGGCACAATAACTTACTTACTCAATGATCAAACCCTTAAAGGTTTAAAATTTGTCGGTGTAGGCTTTGTGACCCCATTTGATGGCATTATCGATGCGGTTACCATTAGTAGCGATGGCATGTTAGTACAACTAGTCGATTTAGATAAAACACCAGGAACCACAAAGTTCCAATTTGTATTAAGTAATACAGCTAATACATTACTCGTTTTAAGTCCAGATCCTCAAATTATCAATCGCCCACAAAAC

SV40 promoter GTGTGTCAGTTAGGGTGTGGAAAGTCCCCAGGCTCCCCAGGCAGGCAGAAGTATGCAAAG

CATGCATCTCAATTAGTCAGCAACCAGGTGTGGAAAGTCCCCAGGCTCCCCAGCAGGCAGAAGTATGCAAAGCATGCATCTCAATTAGTCAGCAACCATAGTCCCGCCCCTAACTCCGCCCATCCCGCCCCTAACTCCGCCCAGTTCCGCCCATTCTCCGCCCCATGGCTGACTAATTTTTTTTATTTATGCAGAGGCCGAGGCCGCCTCTGCCTCTGAGCTATTCCAGAAGTAGTGAGGAGGCTTTTTTGGAGGCCTAGGCTTTTGCAAA

1.6. Primer creation conditions

How primers are created for each fragment depends on its distance from or overlap with adjacent fragments. Each condition is outlined below in Supplemental Figure 2. The relevant parameters in the configuration file for modifying the primer creation conditions are “pcr-primer-max-embed-length”, “fragments-min-junction-length”, “fragments-max-junction-length”, and “pcr-buffer-length”.

***
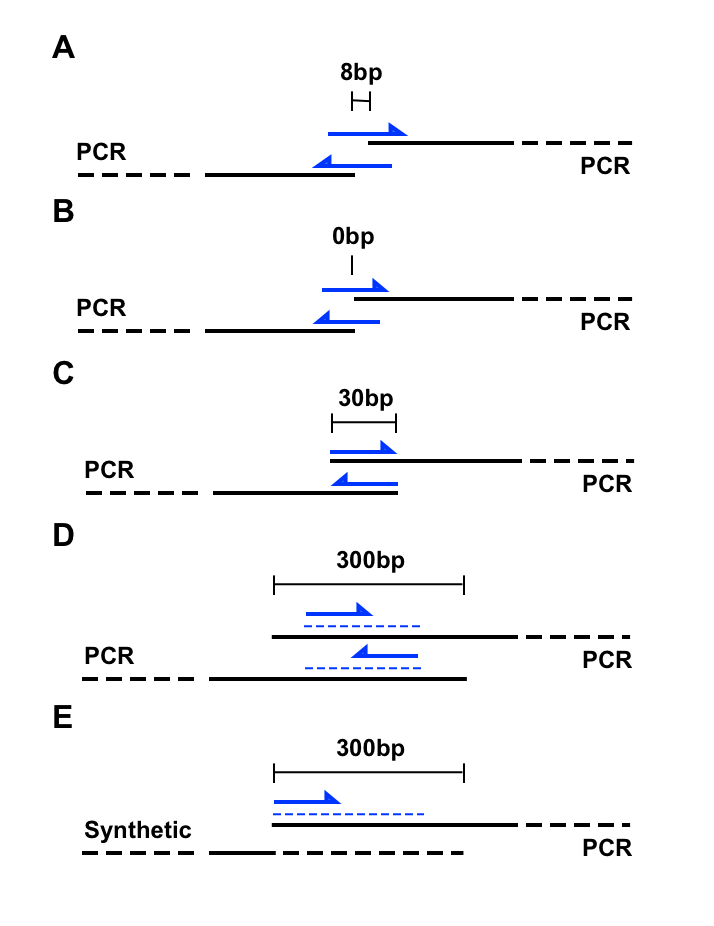
***

***Supplemental Figure B: Primer Creation Conditions***

*Overview of REPP’s approach to primer creation for a fragment given its neighboring fragments distances or overlap. Lengths are in basepairs and are simply representative; thresholds for each condition are specified in the configuration file. a) There is a gap between two repository fragments, relative to their ranges on the reference sequence, that is less than the maximum embed length (pcr-primer-max-embed-length): REPP embeds the reference sequence within the primers and as part of their junction. b) There is no overlap between two repository fragments: REPP creates an overlap with extra bp for the junction within the PCR primers. The overlapping bp for the junction are shared between both fragments. The total overlap after PCR is at least equal to the minimum allowed in the configuration file (fragments-min-junction-length) c) There is sufficient overlap for a junction without modification: REPP creates primers via Primer3 that are fixed at the fragments’ ends. d) There is excessive overlap between two fragments (fragments-max-junction-length): first the left and then the right, Primer3 generates optimal primer pairs for each fragment within a range on the fragment that will reduce the overlap after PCR to something less than the maximum length. e) A repository fragment is adjacent to a synthetic fragment: Primer3 is given flexibility within the start of the fragment to generate an optimal primer pair (pcr-buffer-length), the synthetic fragment is expanded to overlap the post-PCR fragment.*

**2. Commands**

The application, after installation, is available through the “repp” binary – a shortening of “**rep**ository-based **p**lasmid design.” On MacOS/Linux this is in “/usr/local/bin.” On Windows, the binary is installed alongside the rest of the application’s files in “%UserProfile%”; for example: “C:\Users\.repp\repp”.

**2.1. Sequence plasmid specification**

To design a plasmid based on its expected sequence save it to a FASTA or Genbank file. For example:

>2ndVal_mScarlet-I

CAACCTTACCAGAGGGCGCCCCAGCTGGCAATTCCGACGTCTAAGAAACCATTATTATCA...

Then call `repp make sequence` to design it. The following example uses Addgene and a local BLAST database `parts_library.fa` as fragment sources:

repp make sequence --in "./2ndVal_mScarlet-I.fa" --addgene --dbs "parts_library.fa"

REPP includes three embedded databases from large public repositories corresponding to the fragments available in Addgene, DNASU and iGEM. Users can also use their or their lab's fragment databases through the `--dbs` as a list of comma-separated fragment BLAST databases. An example of a plasmid design using Addgene, DNASU, and multiple user-defined BLAST repositories (proteins.fa and backbones.fa) is below:

repp make sequence --in "./2ndVal_mScarlet-I.fa" --addgene --dnasu --dbs "proteins.fa,backbones.fa"

The plasmid sequence in the input file is designed as a circular plasmid by default. In other words, REPP assumes that the sequence includes an insert sequence as well as a backbone. To use the sequence in the input file as an insert sequence but another fragment as a backbone, the --backbone and --enzymes flags are provided. They lookup --backbone in the fragment databases and digest it with the enzyme(s) selected through the --enzymes flag. The linearized backbone will be concatenated to the insert sequence. For example, to insert a GFP_CDS sequence into iGEM's pSB1A3 backbone after linearizing it with PstI and EcoRI:

repp make sequence --in "./GFP_CDS.fa" --addgene --igem --backbone pSB1A3 --enzymes "PstI,EcoRI"

**2.2. Feature plasmid specification**

Feature-based plasmid designs are specified by the list of the features that should be within the outgoing plasmid. Each feature should correspond to a DNA sequence in the feature repository. Users can add additional features to the database through the “set” command (Section 2.5).

Additionally, feature based design can accept the entry IDs of fragments within fragment databases. An example of an iGEM plasmid specification, comprised of multiple BioBrick parts, is below. Each BioBrick ID is used to find the part sequence that it corresponds to using the embedded iGEM database.

repp make features "BBa_R0062,BBa_B0034,BBa_C0040,BBa_B0010,BBa_B0012" --backbone pSB1C3 --enzymes "EcoRI,PstI" --igem

The output file contains the fragments necessary to assembly a plasmid with each of the features as described and the pSB1C3 backbone after digestion with EcoRI and PstI.

**2.3. Fragment plasmid specification**

Fragment-based plasmid designs requires only a multi-FASTA of DNA fragments to assemble in the order they should be pieced together. If adjacent fragments in the assembly lack sufficient end-to-end homology for a junction for Gibson Assembly, additional bp are added via PCR.

An example of an input FASTA with multiple fragments is below (“fragments.fa”):

>gfp

ATGAGTAAAGGAGAAGAACTTTTCACTGGAGTTGTCCCAATTCTTGTTGAATTAGATGGTGATGTTAATGGGCACAAATTTTCTGTCAGTGGAGAGGGTGAAGGTGATGCAACATACGGAAAACTTACCCTTAAATTTATTTGCACTACTGGAAAACTACCTGTTCCATGGCCAACACTTGTCACTACTTTCTCTTATGGTGTTCAATGCTTTTCAAGATACCCAGATCATATGAAACGGCATGACTTTTTCAAGAGTGCCATGCCCGAAGGTTATGTACAGGAAAGAACTATATTTTTCAAAGATGACGGGAACTACAAGACACGTGCTGAAGTCAAGTTTGAAGGTGATACCCTTGTTAATAGAATCGAGTTAAAAGGTATTGATTTTAAAGAAGATGGAAACATTCTTGGACACAAATTGGAATACAACTATAACTCACACAATGTATACATCATGGCAGACAAACAAAAGAATGGAATCAAAGTTAACTTCAAAATTAGACACAACATTGAAGATGGAAGCGTTCAACTAGCAGACCATTATCAACAAAATACTCCAATTGGCGATGGCCCTGTCCTTTTACCAGACAACCATTACCTGTCCACACAATCTGCCCTTTCGAAAGATCCCAACGAAAAGAGAGACCACATGGTCCTTCTTGAGTTTGTAACAGCTGCTGGGATTACACATGGCATGGATGAACTATACAAATAG

>backbone

TACTAGTAGCGGCCGCTGCAGTCCGGCAAAAAAGGGCAAGGTGTCACCACCCTGCCCTTTTTCTTTAAAACCGAAAAGATTACTTCGCGTTATGCAGGCTTCCTCGCTCACTGACTCGCTGCGCTCGGTCGTTCGGCTGCGGCGAGCGGTATCAGCTCACTCAAAGGCGGTAATACGGTTATCCACAGAATCAGGGGATAACGCAGGAAAGAACATGTGAGCAAAAGGCCAGCAAAAGGCCAGGAACCGTAAAAAGGCCGCGTTGCTGGCGTTTTTCCACAGGCTCCGCCCCCCTGACGAGCATCACAAAAATCGACGCTCAAGTCAGAGGTGGCGAAACCCGACAGGACTATAAAGATACCAGGCGTTTCCCCCTGGAAGCTCCCTCGTGCGCTCTCCTGTTCCGACCCTGCCGCTTACCGGATACCTGTCCGCCTTTCTCCCTTCGGGAAGCGTGGCGCTTTCTCATAGCTCACGCTGTAGGTATCTCAGTTCGGTGTAGGTCGTTCGCTCCAAGCTGGGCTGTGTGCACGAACCCCCCGTTCAGCCCGACCGCTGCGCCTTATCCGGTAACTATCGTCTTGAGTCCAACCCGGTAAGACACGACTTATCGCCACTGGCAGCAGCCACTGGTAACAGGATTAGCAGAGCGAGGTATGTAGGCGGTGCTACAGAGTTCTTGAAGTGGTGGCCTAACTACGGCTACACTAGAAGAACAGTATTTGGTATCTGCGCTCTGCTGAAGCCAGTTACCTTCGGAAAAAGAGTTGGTAGCTCTTGATCCGGCAAACAAACCACCGCTGGTAGCGGTGGTTTTTTTGTTTGCAAGCAGCAGATTACGCGCAGAAAAAAAGGATCTCAAGAAGATCCTTTGATCTTTTCTACGGGGTCTGACGCTCAGTGGAACGAAAACTCACGTTAAGGGATTTTGGTCATGAGATTATCAAAAAGGATCTTCACCTAGATCCTTTTAAATTAAAAATGAAGTTTTAAATCAATCTAAAGTATATATGAGTAAACTTGGTCTGACAGCTCGAGGCTTGGATTCTCACCAATAAAAAACGCCCGGCGGCAACCGAGCGTTCTGAACAAATCCAGATGGAGTTCTGAGGTCATTACTGGATCTATCAACAGGAGTCCAAGCGAGCTCGATATCAAATTACGCCCCGCCCTGCCACTCATCGCAGTACTGTTGTAATTCATTAAGCATTCTGCCGACATGGAAGCCATCACAAACGGCATGATGAACCTGAATCGCCAGCGGCATCAGCACCTTGTCGCCTTGCGTATAATATTTGCCCATGGTGAAAACGGGGGCGAAGAAGTTGTCCATATTGGCCACGTTTAAATCAAAACTGGTGAAACTCACCCAGGGATTGGCTGAGACGAAAAACATATTCTCAATAAACCCTTTAGGGAAATAGGCCAGGTTTTCACCGTAACACGCCACATCTTGCGAATATATGTGTAGAAACTGCCGGAAATCGTCGTGGTATTCACTCCAGAGCGATGAAAACGTTTCAGTTTGCTCATGGAAAACGGTGTAACAAGGGTGAACACTATCCCATATCACCAGCTCACCGTCTTTCATTGCCATACGAAATTCCGGATGAGCATTCATCAGGCGGGCAAGAATGTGAATAAAGGCCGGATAAAACTTGTGCTTATTTTTCTTTACGGTCTTTAAAAAGGCCGTAATATCCAGCTGAACGGTCTGGTTATAGGTACATTGAGCAACTGACTGAAATGCCTCAAAATGTTCTTTACGATGCCATTGGGATATATCAACGGTGGTATATCCAGTGATTTTTTTCTCCATTTTAGCTTCCTTAGCTCCTGAAAATCTCGATAACTCAAAAAATACGCCCGGTAGTGATCTTATTTCATTATGGTGAAAGTTGGAACCTCTTACGTGCCCGATCAACTCGAGTGCCACCTGACGTCTAAGAAACCATTATTATCATGACATTAACCTATAAAAATAGGCGTATCACGAGGCAGAATTTCAGATAAAAAAAATCCTTAGCTTTCGCTAAGGATGATTTCTGGAATTCGCGGCCGCTTCTAGAG

And the command to assemble the fragments into the pSB1C3 backbone is as follows:

repp make fragments --in "./fragments.fa"

**2.4. Finding fragments/features/enzymes**

Fragments in BLAST fragment databases, features in the feature database and enzymes in the enzyme database are all queriable via the “find” command. Examples of each, with input command and output, are below:

$ repp ls fragment pSB1C3 --igem

pSB1C3 ~/.repp/igem

TACTAGTAGCGGCCGCTGCAGTCCGGCAAAAAAGGGCAAGGTGTCACCACCCTGCCCTTTTTCTTTAAAACCGAAAAGATTACTTCGCGTTATGCAGGCTTCCTCGCTCACTGACTCGCTGCGCTCGGTCGTTCGGCTGCGGCGAGCGGTATCAGCTCACTCAAAGGCGGTAATACGGTTATCCACAGAATCAGGGGATAACGCAGGAAAGAACATGTGAGCAAAAGGCCAGCAAAAGGCCAGGAACCGTAAAAAGGCCGCGTTGCTGGCGTTTTTCCACAGGCTCCGCCCCCCTGACGAGCATCACAAAAATCGACGCTCAAGTCAGAGGTGGCGAAACCCGACAGGACTATAAAGATACCAGGCGTTTCCC

$ repp ls feature mEGFP

mEGFP AGCAAGGGCGAGGAGCTGTTCACCGGGGTGGTGCCCATCCTGGTCGAGCTGGACGGCGACGTAAACGGCCACAAGTTCAGCGTGCGCGGCGAGGGCGAGGGCGATGCCACCAACGGCAAGCTGACCCTGAAGTTCATCTGCACCACCGGCAAGCTGCCCGTGCCCTGGCCCACCCTCGTGACCACCCTGACC

$ repp ls enzyme Eco

EcoRV GAT^_ATC

Eco53kI GAG^_CTC

EcoO109I RG^GNC_CY

**2.5. Setting feature/enzymes**

Features and enzyme databases are both editable via the command line through REPP. Examples of settings new features and enzymes are below.

$ repp set feature weak_promoter gtgacagctagctcagtcctaggtataatgctagc

$ repp set enzyme BbbI ATG^_CAT

After the above, “weak_promoter” is available as a feature for feature-based plasmid design and BbbI is available as an enzyme for backbone digestion through the “--enzymes" flag.

**3. Test**

**3.1. iGEM**

iGEM test data were accumulated in XML format from the iGEM API page ^14^ and parsed to FASTA format. This FASTA file was converted to a BLAST database using “makeblastdb” and is used as the iGEM database within REPP. We made a copy of the iGEM FASTA file for all years from 2005 to 2018. Each year’s copy excluded all iGEM parts submitted in that year and all years prior. For example, the 2008 iGEM database only had iGEM parts from 2005, 2006, and 2007. Each FASTA file was then parsed to a BLAST databases using the “makeblastdb” binary provided with BLAST ^15^. When building an iGEM plasmid from 2008, it was built using the 2008 BLAST database as a fragment source.

All iGEM parts built by REPP are available in “supp4_igem.txt”. An example of two part entries from the iGEM dataset is below:

>BBa_J06202 2005

tccctatcagtgatagagattgacatccctatcagtgatagagatactgagcactactagagaaagaggagaaatactagatgatcgaactgctgtccgaatccctggaaggtctgtccgctgctatgatcgctgaactgggtcgttaccgtcaccaggttttcatcgaaaaactgggttgggacgttgtttccacctcccgtgttcgtgaccaggagttcgaccagttcgaccacccgcagacccgttacatcgttgctatgtcccgtcagggtatctgcggttgcgctcgtctgctgccgaccaccgacgcttacctgctgaaagacgttttcgcttacctgtgctccgaaaccccgccgtccgacccgtccgtttgggaactgtcccgttacgctgcttccgctgctgacgacccgcagctggctatgaaaatcttctggtcctccctccagtgcgcttggtacctgggtgcttcctccgttgttgctgttaccaccaccgctatggaacgttacttcgttcgtaacggtgttatcctccagcgtctgggtccgccgcagaaagttaaaggtgaaaccctggttgctatctccttcccggcttaccaggaacgtggtctggaaatgctgctgcgttaccacccggaatggctccagggtgttccgctgtccatggctgtttaataatactagagccaggcatcaaataaaacgaaaggctcagtcgaaagactgggcctttcgttttatctgttgtttgtcggtgaacgctctctactagagtcacactggctcaccttcgggtgggcctttctgcgtttata

>BBa_J06503 2005

Atgagcacaaaaaagaaaccattaacacaagagcagcttgaggacgcacgtcgccttaaagcaatttatgaaaaaaagaaaaatgaacttggcttatcccaggaatctgtcgcagacaagatggggatggggcagtcaggcgttggtgctttatttaatggcatcaatgcattaaatgcttataacgccgcattgcttacaaaaattctcaaagttagcgttgaagaatttagcccttcaatcgccagagaaatctacgagatgtatgaagcggttagtatgcagccgtcacttagaagtgagtatgagtaccctgttttttctcatgttcaggcagggatgttctcacctaagcttagaacctttaccaaaggtgatgcggagagatgggtaagcacaaccaaaaaagccagtgattctgcattctggcttgaggttgaaggtaattccatgaccgcaccaacaggctccaagccaagctttcctgacggaatgttaattctcgttgaccctgagcaggctgttgagccaggtgatttctgcatagccagacttgggggtgatgagtttaccttcaagaaactgatcagggatagcggtcaggtgtttttacaaccactaaacccacagtacccaatgatcccatgcaatgagagttgttccgttgtggggaaagttatcgctagtcagtggcctgaagagacgtttggcgctgcaaacgacgaaaactacgctttagtagcttaataacgctgatagtgctagtgtagatcgc

3.1.1. Input

3.1.3. Part count versus cost

REPP returns pareto optimal plasmid designs for each plasmid specification. The reason is that optimal plasmid designs include an optimization of both the number of fragments (minimum) and the dollar cost (minimum). These goals may conflict. There may be a plasmid design with five fragments that is significantly cheaper than another plasmid design with two fragments, one of which is synthetic. Because REPP is selecting pareto optimal plasmid designs, it will never select a plasmid that both costs more and has more fragments than another design. Each design either has fewer fragments that other more expensive designs or has more fragments but is more expensive.

Below is a characterization of plasmid designs where REPP returned multiple plasmid design (pareto optimal designs) for a single plasmid. Each plasmid design’s cost was compared as ratio against the cheapest plasmid design for that plasmid. For example, if two plasmid designs were returned, one with two fragments that is $400 and another with five fragments that is $200, the cost ratio of the two fragments is 2.0 and 1.0, respectively.


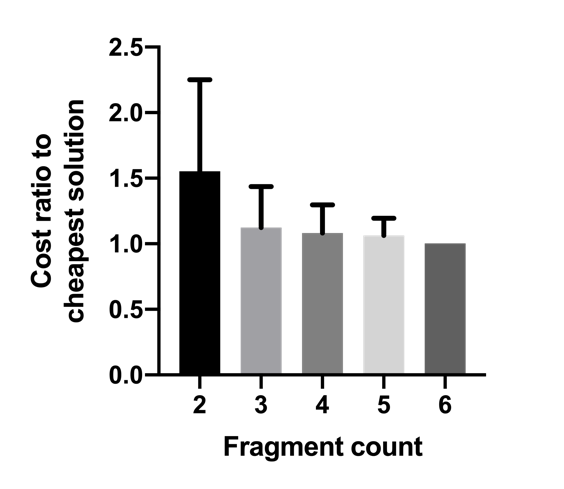


***Supplemental Figure C: Assembly Count Versus Ratio to Cheaper Solutions***

*A comparison of the number of fragments returned in an assembly versus the assembly’s cost ratio to cheaper solutions, for the same target plasmid, with more fragments. Represented as geometric means and bars are geometric standard deviation. Fewer fragment assemblies are preferable for Gibson Assembly but cost more overall. The maximum number of allowed fragments in an assembly was six.*

**3.2. Addgene**

The Addgene dataset was received upon request from Addgene in JSON format. It was parsed to FASTA format. The script used to do so is in the Github repository in “scripts/addgene_fragments_parse.py”. The FASTA file was then converted to a BLAST database using “makeblastdb”. It is the embedded database used when the Addgene flag is provided to a plasmid assembly. All Addgene plasmids from 2018 were separated from the rest of the Addgene FASTA entries and saved to a test dataset to characterize REPP. It is available in “supp5_addgene.txt”. Plasmids from earlier than 2018 were separated into their own BLAST databse, which was referenced via the “--dbs" argument. During the build step, each plasmid in the 2018 dataset was individually loaded, saved to a temporary file, and passed to the “make seq” sub command as documented below.

3.2.1 Input

repp make seq --in ./107133.fa --out ./107133.assembly.json --dbs ./pre-2018-addgene-db.fa --verbose

The input file, “107133.fa”, contains the Addgene plasmid with an ID of 107133. Its full sequence as it existed in the “107133.fa” input file is below. It is accessible from the Addgene repository at <https://www.addgene.org/107133/> and is from a publication by Wang, *et al* ^16^. The database file, “pre-2018-addgene-db.fa,” is the aforementioned BLAST database with all plasmids from 2018 and later removed. The verbose flag is used for progress updates during the plasmid design process and “107133.assembly.json” is the name of the output plasmid design.

107133.fa:

>107133

ACGTTGAGCTGGACGGAAATAGTGGTAAAGTGACATGATTATAGTTTGAAGATTTCTAATTTCACAATTAGAGCAAATGTTGTTCGGTATTTATTTTCAACGGTATTTATACTATTTTCCACCTTTTTCTAGAACATTCGAGCTGCTTGTTGCAAAAGGAGGGCGACTCACATTCGGTACATGGAAAAGTAGTGTACACAATAAAGAGACCCAGATACATTTTCCGTCTGCGTCTCTTTGCACCCACCGGGAGTATTTTCAAACGAATGCATCTAGGACCTTCTAGAACATTCTGTAAGGCTGCAGAATGCGGGTATATAAGGAAAGCGGGCTCAGAGGAAGCCAACACGCTTTGTTCTAGTGCATCTAAAAAACTTCGAAAGGCGCGCCTCTAGAGGATCCCCGGGATTGGCCAAAGGACCCAAAGGTATGTTTCGAATGATACTAACATAACATAGAACATTTTCAGGAGGACCCTTGGCTAGGATGGGCCCTAAAAAGAAGCGTAAAGCTAGCATGAAGCTGTTGTCTTCAATGGAACAAGCTTGCGACATATGCCGGTTAAAAAAACTCAGGTGCTCGAAGGAAAAGCCCAAGTGTGCCAAGTGTCTGAAGAACAACTCGGAGTGCCGTTATTCTCCCAAGGCTATGAGGTCCCCACTGACCAGGGCGCATCTGACAGAGGTAGAATCAAGGTTGGAAAAATTGGAAGATTTGTTTCTGCTCATGTTTCCTCGGGAAAACCTGGACAGCATTTTGAACATGGACTCTTTGGACGAAGTGAAAGTAATGTTGAAACAATTATATATGCAGGACTATGGTGACACGGATGCTGCTATGGATAGCTTGACCCCTATGGAAAATGGCATGTCTGAAGGGCCGAGAAAACACATGAAGACTGCGACATCATCGCCAAAAGTGGATGAAGATGGAAGCCAAAGTCAGTTGAGTGTATCGCCGGAATTCCCGCTGCAGTCTGGTGGCGGAGGGGCTCCTAGGTGCCTCGACCTCAAGACCCAAGTCCAAACCCCACAAGGAATGAAGGAGATCTCCAACATCCAAGTCGGAGACCTCGTCCTCTCCAACACCGGATACAACGAGGTCCTCAACGTCTTCCCAAAGTCCAAGAAGAAGTCCTACAAGATCACCCTCGAGGACGGAAAGGAGATCATCTGCTCCGAGGAGCACCTCTTCCCAACCCAAACCGGAGAGATGAACATCTCCGGAGGACTCAAGGAGGGAATGTGCCTCTACGTCAAGGAGTAAGGTACCGAATTCGCTAGCCGGCCATACAAGTAATCCGGATGATCGACGCCAACGTCGTTGAATTTTCAAATTTTAAATACTGAATATTTGTTTTTTTTCCTATTATTTATTTATTCTCTTTGTGTTTTTTTTCTTGCTTTCTAAAAAATTAATTCAATCCAAATCTAAACATTTTTTTTTCTCTTTCCGTCTCCCAATTCGTATTCCGCTCCTCTCATCTGAACACAATGTGCAAGTTTATTTATCTTCTCGCTTTCATTTCATTAGGACGTGGGGGGAATTGGTGGAAGGGGGAAACACACAAAAGGATGATGGAAATGAAATAAGGACACACAATATGCAACAACATTCAATTCAGAAATATGGAGGAAGGTTTAAAAGAAAACATAAAAATATATAGAGGAGGAAGGAAAACTAGTAAAAAATAAGCAAAGAAATTAGGCGAACGATGAGAATTGTCCTCGCTTGGGCCCAAAAGGCCTTTTTTTTTTGGCGCCGACGTCAGGTGGCACTTTTCGGGGAAATGTGCGCGGAACCCCTATTTGTTTATTTTTCTAAATACATTCAAATATGTATCCGCTCATGAGACAATAACCCTGATAAATGCTTCAATAATATTGAAAAAGGAAGAGTATGAGTATTCAACATTTCCGTGTCGCCCTTATTCCCTTTTTTGCGGCATTTTGCCTTCCTGTTTTTGCTCACCCAGAAACGCTGGTGAAAGTAAAAGATGCTGAAGATCAGTTGGGTGCACGAGTGGGTTACATCGAACTGGATCTCAACAGCGGTAAGATCCTTGAGAGTTTTCGCCCCGAAGAACGTTTTCCAATGATGAGCACTTTTAAAGTTCTGCTATGTGGCGCGGTATTATCCCGTATTGACGCCGGGCAAGAGCAACTCGGTCGCCGCATACACTATTCTCAGAATGACTTGGTTGAGTACTCACCAGTCACAGAAAAGCATCTTACGGATGGCATGACAGTAAGAGAATTATGCAGTGCTGCCATAACCATGAGTGATAACACTGCGGCCAACTTACTTCTGACAACGATCGGAGGACCGAAGGAGCTAACCGCTTTTTTGCACAACATGGGGGATCATGTAACTCGCCTTGATCGTTGGGAACCGGAGCTGAATGAAGCCATACCAAACGACGAGCGTGACACCACGATGCCTGTAGCAATGGCAACAACGTTGCGCAAACTATTAACTGGCGAACTACTTACTCTAGCTTCCCGGCAACAATTAATAGACTGGATGGAGGCGGATAAAGTTGCAGGACCACTTCTGCGCTCGGCCCTTCCGGCTGGCTGGTTTATTGCTGATAAATCTGGAGCCGGTGAGCGTGGGTCTCGCGGTATCATTGCAGCACTGGGGCCAGATGGTAAGCCCTCCCGTATCGTAGTTATCTACACGACGGGGAGTCAGGCAACTATGGATGAACGAAATAGACAGATCGCTGAGATAGGTGCCTCACTGATTAAGCATTGGTAACTGTCAGACCAAGTTTACTCATATATACTTTAGATTGATTTAAAACTTCATTTTTAATTTAAAAGGATCTAGGTGAAGATCCTTTTTGATAATCTCATGACCAAAATCCCTTAACGTGAGTTTTCGTTCCACTGAGCGTCAGACCCCGTAGAAAAGATCAAAGGATCTTCTTGAGATCCTTTTTTTCTGCGCGTAATCTGCTGCTTGCAAACAAAAAAACCACCGCTACCAGCGGTGGTTTGTTTGCCGGATCAAGAGCTACCAACTCTTTTTCCGAAGGTAACTGGCTTCAGCAGAGCGCAGATACCAAATACTGTTCTTCTAGTGTAGCCGTAGTTAGGCCACCACTTCAAGAACTCTGTAGCACCGCCTACATACCTCGCTCTGCTAATCCTGTTACCAGTGGCTGCTGCCAGTGGCGATAAGTCGTGTCTTACCGGGTTGGACTCAAGACGATAGTTACCGGATAAGGCGCAGCGGTCGGGCTGAACGGGGGGTTCGTGCACACAGCCCAGCTTGGAGCGAACGACCTACACCGAACTGAGATACCTACAGCGTGAGCTATGAGAAAGCGCCACGCTTCCCGAAGGGAGAAAGGCGGACAGGTATCCGGTAAGCGGCAGGGTCGGAACAGGAGAGCGCACGAGGGAGCTTCCAGGGGGAAACGCCTGGTATCTTTATAGTCCTGTCGGGTTTCGCCACCTCTGACTTGAGCGTCGATTTTTGTGATGCTCGTCAGGGGGGCGGAGCCTATGGAAAAACGCCAGCAACGCGGCCTTTTTACGGTTCCTGGCCTTTTGCTGGCCTTTTGCTCACATGTTCTTTCCTGCGTTATCCCCTGATTCTGTGGATAACCGTATTACCGCCTTTGAGTGAGCTGATACCGCTCGCCGCAGCCGAACGACCGAGCGCAGCGAGTCAGTGAGCGAGGAAGCGGAAGAGCGCCCAATACGCAAACCGCCTCTCCCCGCGCGTTGGCCGATTCATTAATGCAGCTGGCACGACAGGTTTCCCGACTGGAAAGCGGGCAGTGAGCGCAACGCAATTAATGTGAGTTAGCTCACTCATTAGGCACCCCAGGCTTTACACTTTATGCTTCCGGCTCGTATGTTGTGTGGAATTGTGAGCGGATAACAATTTCACACAGGAAACAGCTATGACCATGATTACGCCAAGCTGTAAGTTTAAACATGATCTTACTAACTAACTATTCTCATTTAAATTTTCAGAGCTTAAAAATGGCTGAAATCACTCACAACGATGGATACGCTAACAACTTGGAAATGAAATAAGCTTGCATGCGCGGCCGCACTGACTGGGCCGGCC

3.2.2 Output

107133.output.json

{

"target": "107133",

"seq": "ACGTTGAGCTGGACGGAAATAGTGGTAAAGTGACATGATTATAGTTTGAAGATTTCTAATTTCACAATTAGAGCAAATGTTGTTCGGTATTTATTTTCAACGGTATTTATACTATTTTCCACCTTTTTCTAGAACATTCGAGCTGCTTGTTGCAAAAGGAGGGCGACTCACATTCGGTACATGGAAAAGTAGTGTACACAATAAAGAGACCCAGATACATTTTCCGTCTGCGTCTCTTTGCACCCACCGGGAGTATTTTCAAACGAATGCATCTAGGACCTTCTAGAACATTCTGTAAGGCTGCAGAATGCGGGTATATAAGGAAAGCGGGCTCAGAGGAAGCCAACACGCTTTGTTCTAGTGCATCTAAAAAACTTCGAAAGGCGCGCCTCTAGAGGATCCCCGGGATTGGCCAAAGGACCCAAAGGTATGTTTCGAATGATACTAACATAACATAGAACATTTTCAGGAGGACCCTTGGCTAGGATGGGCCCTAAAAAGAAGCGTAAAGCTAGCATGAAGCTGTTGTCTTCAATGGAACAAGCTTGCGACATATGCCGGTTAAAAAAACTCAGGTGCTCGAAGGAAAAGCCCAAGTGTGCCAAGTGTCTGAAGAACAACTCGGAGTGCCGTTATTCTCCCAAGGCTATGAGGTCCCCACTGACCAGGGCGCATCTGACAGAGGTAGAATCAAGGTTGGAAAAATTGGAAGATTTGTTTCTGCTCATGTTTCCTCGGGAAAACCTGGACAGCATTTTGAACATGGACTCTTTGGACGAAGTGAAAGTAATGTTGAAACAATTATATATGCAGGACTATGGTGACACGGATGCTGCTATGGATAGCTTGACCCCTATGGAAAATGGCATGTCTGAAGGGCCGAGAAAACACATGAAGACTGCGACATCATCGCCAAAAGTGGATGAAGATGGAAGCCAAAGTCAGTTGAGTGTATCGCCGGAATTCCCGCTGCAGTCTGGTGGCGGAGGGGCTCCTAGGTGCCTCGACCTCAAGACCCAAGTCCAAACCCCACAAGGAATGAAGGAGATCTCCAACATCCAAGTCGGAGACCTCGTCCTCTCCAACACCGGATACAACGAGGTCCTCAACGTCTTCCCAAAGTCCAAGAAGAAGTCCTACAAGATCACCCTCGAGGACGGAAAGGAGATCATCTGCTCCGAGGAGCACCTCTTCCCAACCCAAACCGGAGAGATGAACATCTCCGGAGGACTCAAGGAGGGAATGTGCCTCTACGTCAAGGAGTAAGGTACCGAATTCGCTAGCCGGCCATACAAGTAATCCGGATGATCGACGCCAACGTCGTTGAATTTTCAAATTTTAAATACTGAATATTTGTTTTTTTTCCTATTATTTATTTATTCTCTTTGTGTTTTTTTTCTTGCTTTCTAAAAAATTAATTCAATCCAAATCTAAACATTTTTTTTTCTCTTTCCGTCTCCCAATTCGTATTCCGCTCCTCTCATCTGAACACAATGTGCAAGTTTATTTATCTTCTCGCTTTCATTTCATTAGGACGTGGGGGGAATTGGTGGAAGGGGGAAACACACAAAAGGATGATGGAAATGAAATAAGGACACACAATATGCAACAACATTCAATTCAGAAATATGGAGGAAGGTTTAAAAGAAAACATAAAAATATATAGAGGAGGAAGGAAAACTAGTAAAAAATAAGCAAAGAAATTAGGCGAACGATGAGAATTGTCCTCGCTTGGGCCCAAAAGGCCTTTTTTTTTTGGCGCCGACGTCAGGTGGCACTTTTCGGGGAAATGTGCGCGGAACCCCTATTTGTTTATTTTTCTAAATACATTCAAATATGTATCCGCTCATGAGACAATAACCCTGATAAATGCTTCAATAATATTGAAAAAGGAAGAGTATGAGTATTCAACATTTCCGTGTCGCCCTTATTCCCTTTTTTGCGGCATTTTGCCTTCCTGTTTTTGCTCACCCAGAAACGCTGGTGAAAGTAAAAGATGCTGAAGATCAGTTGGGTGCACGAGTGGGTTACATCGAACTGGATCTCAACAGCGGTAAGATCCTTGAGAGTTTTCGCCCCGAAGAACGTTTTCCAATGATGAGCACTTTTAAAGTTCTGCTATGTGGCGCGGTATTATCCCGTATTGACGCCGGGCAAGAGCAACTCGGTCGCCGCATACACTATTCTCAGAATGACTTGGTTGAGTACTCACCAGTCACAGAAAAGCATCTTACGGATGGCATGACAGTAAGAGAATTATGCAGTGCTGCCATAACCATGAGTGATAACACTGCGGCCAACTTACTTCTGACAACGATCGGAGGACCGAAGGAGCTAACCGCTTTTTTGCACAACATGGGGGATCATGTAACTCGCCTTGATCGTTGGGAACCGGAGCTGAATGAAGCCATACCAAACGACGAGCGTGACACCACGATGCCTGTAGCAATGGCAACAACGTTGCGCAAACTATTAACTGGCGAACTACTTACTCTAGCTTCCCGGCAACAATTAATAGACTGGATGGAGGCGGATAAAGTTGCAGGACCACTTCTGCGCTCGGCCCTTCCGGCTGGCTGGTTTATTGCTGATAAATCTGGAGCCGGTGAGCGTGGGTCTCGCGGTATCATTGCAGCACTGGGGCCAGATGGTAAGCCCTCCCGTATCGTAGTTATCTACACGACGGGGAGTCAGGCAACTATGGATGAACGAAATAGACAGATCGCTGAGATAGGTGCCTCACTGATTAAGCATTGGTAACTGTCAGACCAAGTTTACTCATATATACTTTAGATTGATTTAAAACTTCATTTTTAATTTAAAAGGATCTAGGTGAAGATCCTTTTTGATAATCTCATGACCAAAATCCCTTAACGTGAGTTTTCGTTCCACTGAGCGTCAGACCCCGTAGAAAAGATCAAAGGATCTTCTTGAGATCCTTTTTTTCTGCGCGTAATCTGCTGCTTGCAAACAAAAAAACCACCGCTACCAGCGGTGGTTTGTTTGCCGGATCAAGAGCTACCAACTCTTTTTCCGAAGGTAACTGGCTTCAGCAGAGCGCAGATACCAAATACTGTTCTTCTAGTGTAGCCGTAGTTAGGCCACCACTTCAAGAACTCTGTAGCACCGCCTACATACCTCGCTCTGCTAATCCTGTTACCAGTGGCTGCTGCCAGTGGCGATAAGTCGTGTCTTACCGGGTTGGACTCAAGACGATAGTTACCGGATAAGGCGCAGCGGTCGGGCTGAACGGGGGGTTCGTGCACACAGCCCAGCTTGGAGCGAACGACCTACACCGAACTGAGATACCTACAGCGTGAGCTATGAGAAAGCGCCACGCTTCCCGAAGGGAGAAAGGCGGACAGGTATCCGGTAAGCGGCAGGGTCGGAACAGGAGAGCGCACGAGGGAGCTTCCAGGGGGAAACGCCTGGTATCTTTATAGTCCTGTCGGGTTTCGCCACCTCTGACTTGAGCGTCGATTTTTGTGATGCTCGTCAGGGGGGCGGAGCCTATGGAAAAACGCCAGCAACGCGGCCTTTTTACGGTTCCTGGCCTTTTGCTGGCCTTTTGCTCACATGTTCTTTCCTGCGTTATCCCCTGATTCTGTGGATAACCGTATTACCGCCTTTGAGTGAGCTGATACCGCTCGCCGCAGCCGAACGACCGAGCGCAGCGAGTCAGTGAGCGAGGAAGCGGAAGAGCGCCCAATACGCAAACCGCCTCTCCCCGCGCGTTGGCCGATTCATTAATGCAGCTGGCACGACAGGTTTCCCGACTGGAAAGCGGGCAGTGAGCGCAACGCAATTAATGTGAGTTAGCTCACTCATTAGGCACCCCAGGCTTTACACTTTATGCTTCCGGCTCGTATGTTGTGTGGAATTGTGAGCGGATAACAATTTCACACAGGAAACAGCTATGACCATGATTACGCCAAGCTGTAAGTTTAAACATGATCTTACTAACTAACTATTCTCATTTAAATTTTCAGAGCTTAAAAATGGCTGAAATCACTCACAACGATGGATACGCTAACAACTTGGAAATGAAATAAGCTTGCATGCGCGGCCGCACTGACTGGGCCGGCC",

"time": "2019/06/06 19:40:42",

"execution": 23.854875237999998,

"solutions": [

{

"count": 2,

"cost": 351.25,

"fragments": [

{

"type": "pcr",

"cost": 89.27,

"url": "https://www.addgene.org/85583/",

"seq": "TAAGGTACCGAATTCGCTAGCCGGCCATACAAGTAATCCGGATGATCGACGCCAACGTCGTTGAATTTTCAAATTTTAAATACTGAATATTTGTTTTTTTTCCTATTATTTATTTATTCTCTTTGTGTTTTTTTTCTTGCTTTCTAAAAAATTAATTCAATCCAAATCTAAACATTTTTTTTTCTCTTTCCGTCTCCCAATTCGTATTCCGCTCCTCTCATCTGAACACAATGTGCAAGTTTATTTATCTTCTCGCTTTCATTTCATTAGGACGTGGGGGGAATTGGTGGAAGGGGGAAACACACAAAAGGATGATGGAAATGAAATAAGGACACACAATATGCAACAACATTCAATTCAGAAATATGGAGGAAGGTTTAAAAGAAAACATAAAAATATATAGAGGAGGAAGGAAAACTAGTAAAAAATAAGCAAAGAAATTAGGCGAACGATGAGAATTGTCCTCGCTTGGGCCCAAAAGGCCTTTTTTTTTTGGCGCCGACGTCAGGTGGCACTTTTCGGGGAAATGTGCGCGGAACCCCTATTTGTTTATTTTTCTAAATACATTCAAATATGTATCCGCTCATGAGACAATAACCCTGATAAATGCTTCAATAATATTGAAAAAGGAAGAGTATGAGTATTCAACATTTCCGTGTCGCCCTTATTCCCTTTTTTGCGGCATTTTGCCTTCCTGTTTTTGCTCACCCAGAAACGCTGGTGAAAGTAAAAGATGCTGAAGATCAGTTGGGTGCACGAGTGGGTTACATCGAACTGGATCTCAACAGCGGTAAGATCCTTGAGAGTTTTCGCCCCGAAGAACGTTTTCCAATGATGAGCACTTTTAAAGTTCTGCTATGTGGCGCGGTATTATCCCGTATTGACGCCGGGCAAGAGCAACTCGGTCGCCGCATACACTATTCTCAGAATGACTTGGTTGAGTACTCACCAGTCACAGAAAAGCATCTTACGGATGGCATGACAGTAAGAGAATTATGCAGTGCTGCCATAACCATGAGTGATAACACTGCGGCCAACTTACTTCTGACAACGATCGGAGGACCGAAGGAGCTAACCGCTTTTTTGCACAACATGGGGGATCATGTAACTCGCCTTGATCGTTGGGAACCGGAGCTGAATGAAGCCATACCAAACGACGAGCGTGACACCACGATGCCTGTAGCAATGGCAACAACGTTGCGCAAACTATTAACTGGCGAACTACTTACTCTAGCTTCCCGGCAACAATTAATAGACTGGATGGAGGCGGATAAAGTTGCAGGACCACTTCTGCGCTCGGCCCTTCCGGCTGGCTGGTTTATTGCTGATAAATCTGGAGCCGGTGAGCGTGGGTCTCGCGGTATCATTGCAGCACTGGGGCCAGATGGTAAGCCCTCCCGTATCGTAGTTATCTACACGACGGGGAGTCAGGCAACTATGGATGAACGAAATAGACAGATCGCTGAGATAGGTGCCTCACTGATTAAGCATTGGTAACTGTCAGACCAAGTTTACTCATATATACTTTAGATTGATTTAAAACTTCATTTTTAATTTAAAAGGATCTAGGTGAAGATCCTTTTTGATAATCTCATGACCAAAATCCCTTAACGTGAGTTTTCGTTCCACTGAGCGTCAGACCCCGTAGAAAAGATCAAAGGATCTTCTTGAGATCCTTTTTTTCTGCGCGTAATCTGCTGCTTGCAAACAAAAAAACCACCGCTACCAGCGGTGGTTTGTTTGCCGGATCAAGAGCTACCAACTCTTTTTCCGAAGGTAACTGGCTTCAGCAGAGCGCAGATACCAAATACTGTTCTTCTAGTGTAGCCGTAGTTAGGCCACCACTTCAAGAACTCTGTAGCACCGCCTACATACCTCGCTCTGCTAATCCTGTTACCAGTGGCTGCTGCCAGTGGCGATAAGTCGTGTCTTACCGGGTTGGACTCAAGACGATAGTTACCGGATAAGGCGCAGCGGTCGGGCTGAACGGGGGGTTCGTGCACACAGCCCAGCTTGGAGCGAACGACCTACACCGAACTGAGATACCTACAGCGTGAGCTATGAGAAAGCGCCACGCTTCCCGAAGGGAGAAAGGCGGACAGGTATCCGGTAAGCGGCAGGGTCGGAACAGGAGAGCGCACGAGGGAGCTTCCAGGGGGAAACGCCTGGTATCTTTATAGTCCTGTCGGGTTTCGCCACCTCTGACTTGAGCGTCGATTTTTGTGATGCTCGTCAGGGGGGCGGAGCCTATGGAAAAACGCCAGCAACGCGGCCTTTTTACGGTTCCTGGCCTTTTGCTGGCCTTTTGCTCACATGTTCTTTCCTGCGTTATCCCCTGATTCTGTGGATAACCGTATTACCGCCTTTGAGTGAGCTGATACCGCTCGCCGCAGCCGAACGACCGAGCGCAGCGAGTCAGTGAGCGAGGAAGCGGAAGAGCGCCCAATACGCAAACCGCCTCTCCCCGCGCGTTGGCCGATTCATTAATGCAGCTGGCACGACAGGTTTCCCGACTGGAAAGCGGGCAGTGAGCGCAACGCAATTAATGTGAGTTAGCTCACTCATTAGGCACCCCAGGCTTTACACTTTATGCTTCCGGCTCGTATGTTGTGTGGAATTGTGAGCGGATAACAATTTCACACAGGAAACAGCTATGACCATGATTACGCCAAGCTGTAAGTTTAAACATGATCTTACTAACTAACTATTCTCATTTAAATTTTCAGAGCTTAAAAATGGCTGAAATCACTCACAACGATGGATACGCTAACAACTTGGAAATGAAATAAGCTTGCATGCGCGGCCGCACTGACTGGGCCGGCC",

"pcrSeq": "TAAGGTACCGAATTCGCTAGCCGGCCATACAAGTAATCCGGATGATCGACGCCAACGTCGTTGAATTTTCAAATTTTAAATACTGAATATTTGTTTTTTTTCCTATTATTTATTTATTCTCTTTGTGTTTTTTTTCTTGCTTTCTAAAAAATTAATTCAATCCAAATCTAAACATTTTTTTTTCTCTTTCCGTCTCCCAATTCGTATTCCGCTCCTCTCATCTGAACACAATGTGCAAGTTTATTTATCTTCTCGCTTTCATTTCATTAGGACGTGGGGGGAATTGGTGGAAGGGGGAAACACACAAAAGGATGATGGAAATGAAATAAGGACACACAATATGCAACAACATTCAATTCAGAAATATGGAGGAAGGTTTAAAAGAAAACATAAAAATATATAGAGGAGGAAGGAAAACTAGTAAAAAATAAGCAAAGAAATTAGGCGAACGATGAGAATTGTCCTCGCTTGGGCCCAAAAGGCCTTTTTTTTTTGGCGCCGACGTCAGGTGGCACTTTTCGGGGAAATGTGCGCGGAACCCCTATTTGTTTATTTTTCTAAATACATTCAAATATGTATCCGCTCATGAGACAATAACCCTGATAAATGCTTCAATAATATTGAAAAAGGAAGAGTATGAGTATTCAACATTTCCGTGTCGCCCTTATTCCCTTTTTTGCGGCATTTTGCCTTCCTGTTTTTGCTCACCCAGAAACGCTGGTGAAAGTAAAAGATGCTGAAGATCAGTTGGGTGCACGAGTGGGTTACATCGAACTGGATCTCAACAGCGGTAAGATCCTTGAGAGTTTTCGCCCCGAAGAACGTTTTCCAATGATGAGCACTTTTAAAGTTCTGCTATGTGGCGCGGTATTATCCCGTATTGACGCCGGGCAAGAGCAACTCGGTCGCCGCATACACTATTCTCAGAATGACTTGGTTGAGTACTCACCAGTCACAGAAAAGCATCTTACGGATGGCATGACAGTAAGAGAATTATGCAGTGCTGCCATAACCATGAGTGATAACACTGCGGCCAACTTACTTCTGACAACGATCGGAGGACCGAAGGAGCTAACCGCTTTTTTGCACAACATGGGGGATCATGTAACTCGCCTTGATCGTTGGGAACCGGAGCTGAATGAAGCCATACCAAACGACGAGCGTGACACCACGATGCCTGTAGCAATGGCAACAACGTTGCGCAAACTATTAACTGGCGAACTACTTACTCTAGCTTCCCGGCAACAATTAATAGACTGGATGGAGGCGGATAAAGTTGCAGGACCACTTCTGCGCTCGGCCCTTCCGGCTGGCTGGTTTATTGCTGATAAATCTGGAGCCGGTGAGCGTGGGTCTCGCGGTATCATTGCAGCACTGGGGCCAGATGGTAAGCCCTCCCGTATCGTAGTTATCTACACGACGGGGAGTCAGGCAACTATGGATGAACGAAATAGACAGATCGCTGAGATAGGTGCCTCACTGATTAAGCATTGGTAACTGTCAGACCAAGTTTACTCATATATACTTTAGATTGATTTAAAACTTCATTTTTAATTTAAAAGGATCTAGGTGAAGATCCTTTTTGATAATCTCATGACCAAAATCCCTTAACGTGAGTTTTCGTTCCACTGAGCGTCAGACCCCGTAGAAAAGATCAAAGGATCTTCTTGAGATCCTTTTTTTCTGCGCGTAATCTGCTGCTTGCAAACAAAAAAACCACCGCTACCAGCGGTGGTTTGTTTGCCGGATCAAGAGCTACCAACTCTTTTTCCGAAGGTAACTGGCTTCAGCAGAGCGCAGATACCAAATACTGTTCTTCTAGTGTAGCCGTAGTTAGGCCACCACTTCAAGAACTCTGTAGCACCGCCTACATACCTCGCTCTGCTAATCCTGTTACCAGTGGCTGCTGCCAGTGGCGATAAGTCGTGTCTTACCGGGTTGGACTCAAGACGATAGTTACCGGATAAGGCGCAGCGGTCGGGCTGAACGGGGGGTTCGTGCACACAGCCCAGCTTGGAGCGAACGACCTACACCGAACTGAGATACCTACAGCGTGAGCTATGAGAAAGCGCCACGCTTCCCGAAGGGAGAAAGGCGGACAGGTATCCGGTAAGCGGCAGGGTCGGAACAGGAGAGCGCACGAGGGAGCTTCCAGGGGGAAACGCCTGGTATCTTTATAGTCCTGTCGGGTTTCGCCACCTCTGACTTGAGCGTCGATTTTTGTGATGCTCGTCAGGGGGGCGGAGCCTATGGAAAAACGCCAGCAACGCGGCCTTTTTACGGTTCCTGGCCTTTTGCTGGCCTTTTGCTCACATGTTCTTTCCTGCGTTATCCCCTGATTCTGTGGATAACCGTATTACCGCCTTTGAGTGAGCTGATACCGCTCGCCGCAGCCGAACGACCGAGCGCAGCGAGTCAGTGAGCGAGGAAGCGGAAGAGCGCCCAATACGCAAACCGCCTCTCCCCGCGCGTTGGCCGATTCATTAATGCAGCTGGCACGACAGGTTTCCCGACTGGAAAGCGGGCAGTGAGCGCAACGCAATTAATGTGAGTTAGCTCACTCATTAGGCACCCCAGGCTTTACACTTTATGCTTCCGGCTCGTATGTTGTGTGGAATTGTGAGCGGATAACAATTTCACACAGGAAACAGCTATGACCATGATTACGCCAAGCTGTAAGTTTAAACATGATCTTACTAACTAACTATTCTCATTTAAATTTTCAGAGCTTAAAAATGGCTGAAATCACTCACAACGATGGATACGCTAACAACTTGGAAATGAAATAAGCTTGCATGCGCGGCCGCACTGACTGGGCCGGCC",

"primers": [

{

"seq": "TAAGGTACCGAATTCGCTAGCC",

"strand": true,

"penalty": 2.035066,

"pairPenalty": 10.128466,

"tm": 59.965,

"gc": 50

},

{

"seq": "GGCCGGCCCAGTCAGTGC",

"strand": false,

"penalty": 8.0934,

"pairPenalty": 10.128466,

"tm": 66.093,

"gc": 77.778

}

]

},

{

"id": "85583-85583-synthesis-1",

"type": "synthetic",

"cost": 249,

"seq": "ACTGACTGGGCCGGCCACGTTGAGCTGGACGGAAATAGTGGTAAAGTGACATGATTATAGTTTGAAGATTTCTAATTTCACAATTAGAGCAAATGTTGTTCGGTATTTATTTTCAACGGTATTTATACTATTTTCCACCTTTTTCTAGAACATTCGAGCTGCTTGTTGCAAAAGGAGGGCGACTCACATTCGGTACATGGAAAAGTAGTGTACACAATAAAGAGACCCAGATACATTTTCCGTCTGCGTCTCTTTGCACCCACCGGGAGTATTTTCAAACGAATGCATCTAGGACCTTCTAGAACATTCTGTAAGGCTGCAGAATGCGGGTATATAAGGAAAGCGGGCTCAGAGGAAGCCAACACGCTTTGTTCTAGTGCATCTAAAAAACTTCGAAAGGCGCGCCTCTAGAGGATCCCCGGGATTGGCCAAAGGACCCAAAGGTATGTTTCGAATGATACTAACATAACATAGAACATTTTCAGGAGGACCCTTGGCTAGGATGGGCCCTAAAAAGAAGCGTAAAGCTAGCATGAAGCTGTTGTCTTCAATGGAACAAGCTTGCGACATATGCCGGTTAAAAAAACTCAGGTGCTCGAAGGAAAAGCCCAAGTGTGCCAAGTGTCTGAAGAACAACTCGGAGTGCCGTTATTCTCCCAAGGCTATGAGGTCCCCACTGACCAGGGCGCATCTGACAGAGGTAGAATCAAGGTTGGAAAAATTGGAAGATTTGTTTCTGCTCATGTTTCCTCGGGAAAACCTGGACAGCATTTTGAACATGGACTCTTTGGACGAAGTGAAAGTAATGTTGAAACAATTATATATGCAGGACTATGGTGACACGGATGCTGCTATGGATAGCTTGACCCCTATGGAAAATGGCATGTCTGAAGGGCCGAGAAAACACATGAAGACTGCGACATCATCGCCAAAAGTGGATGAAGATGGAAGCCAAAGTCAGTTGAGTGTATCGCCGGAATTCCCGCTGCAGTCTGGTGGCGGAGGGGCTCCTAGGTGCCTCGACCTCAAGACCCAAGTCCAAACCCCACAAGGAATGAAGGAGATCTCCAACATCCAAGTCGGAGACCTCGTCCTCTCCAACACCGGATACAACGAGGTCCTCAACGTCTTCCCAAAGTCCAAGAAGAAGTCCTACAAGATCACCCTCGAGGACGGAAAGGAGATCATCTGCTCCGAGGAGCACCTCTTCCCAACCCAAACCGGAGAGATGAACATCTCCGGAGGACTCAAGGAGGGAATGTGCCTCTACGTCAAGGAGTAAGGTACCGAATTCG"

}

]

}

]

}

**References**

1. Rational Design of High-Number dsDNA Fragments Based on Thermodynamics for the Construction of Full-Length Genes in a Single Reaction. Available at: https://www.ncbi.nlm.nih.gov/pmc/articles/PMC4696799/. (Accessed: 18th June 2019)

2. Untergasser, A. *et al.* Primer3—new capabilities and interfaces. *Nucleic Acids Res.* **40**, e115 (2012).

3. Gibson Assembly® Master Mix | NEB. Available at: https://www.neb.com/products/e2611-gibson-assembly-master-mix#Product%20Information. (Accessed: 18th June 2019)

4. Custom DNA oligo products. Available at: https://www.idtdna.com/pages/products/custom-dna-rna/dna-oligos/custom-dna-oligos. (Accessed: 18th June 2019)

5. Taq DNA Polymerase PCR Buffer (10X) - Thermo Fisher Scientific. Available at: https://www.thermofisher.com/order/catalog/product/18067017. (Accessed: 18th June 2019)

6. gBlocks Gene Fragments. Available at: https://www.idtdna.com/pages/products/genes-and-gene-fragments/gblocks-gene-fragments. (Accessed: 18th June 2019)

7. Custom gene synthesis. Available at: https://www.idtdna.com/pages/products/genes-and-gene-fragments/custom-gene-synthesis. (Accessed: 18th June 2019)

8. Addgene: How to Order. Available at: https://www.addgene.org/ordering/. (Accessed: 18th June 2019)

9. Catalog - parts.igem.org. Available at: http://parts.igem.org/Catalog. (Accessed: 18th June 2019)

10. Seiler, C. Y. *et al.* DNASU plasmid and PSI: Biology-Materials repositories: resources to accelerate biological research. *Nucleic Acids Res.* **42**, D1253–D1260 (2013).

11. Information, N. C. for B., Pike, U. S. N. L. of M. 8600 R., MD, B. & Usa, 20894. *Appendices*. (National Center for Biotechnology Information (US), 2018).

12. Alphabetized List of Recognition Specificities | NEB. Available at: https://www.neb.com/tools-and-resources/selection-charts/alphabetized-list-of-recognition-specificities. (Accessed: 18th June 2019)

13. SnapGene | Software for everyday molecular biology. *SnapGene* Available at: https://www.snapgene.com/. (Accessed: 30th April 2019)

14. Registry API - parts.igem.org. Available at: http://parts.igem.org/Registry_API. (Accessed: 23rd April 2019)

15. Information, N. C. for B., Pike, U. S. N. L. of M. 8600 R., MD, B. & Usa, 20894. *Building a BLAST database with local sequences*. (National Center for Biotechnology Information (US), 2008).

16. Wang, H., Liu, J., Yuet, K. P., Hill, A. J. & Sternberg, P. W. Split cGAL, an intersectional strategy using a split intein for refined spatiotemporal transgene control in Caenorhabditis elegans. *Proc. Natl. Acad. Sci. U. S. A.* **115**, 3900–3905 (2018).
